# Modulating DNA Repair Pathways to Diversify Genomic Alterations in Saccharomyces cerevisiae

**DOI:** 10.1128/spectrum.02326-21

**Published:** 2022-03-30

**Authors:** Zhen Wang, Yuping Lin, Zongjie Dai, Qinhong Wang

**Affiliations:** a CAS Key Laboratory of Systems Microbial Biotechnology, Tianjin Institute of Industrial Biotechnology, Chinese Academy of Sciences, Tianjin, China; b College of Science & Technology, Hebei Agricultural University, Cangzhou, Hebei, China; c National Center of Technology Innovation for Synthetic Biology, Tianjin, China; The Ohio State University

**Keywords:** DSB repair, diversified mutation, gene expression, genome editing, mutational efficiency

## Abstract

Nuclease based genome editing systems have emerged as powerful tools to drive genomic alterations and enhance genome evolution via precise engineering in the various human and microbial cells. However, error-prone DNA repair has not been well studied previously to generate diverse genomic alterations and novel phenotypes. Here, we systematically investigated the potential interplay between DNA double strand break (DSB) repair and genome editing tools, and found that modulating the DSB end resection proteins could significantly improve mutational efficiency and diversity without exogenous DNA template in yeast. Deleting *SAE2*, *EXO1*, or *FUN30*, or overexpressing *MRE11*-H125N (nuclease-dead allele of *MRE11*), for DSB end resection markedly increased the efficiency of CRISPR/SpCas9 (more than 22-fold) and CRISPR/AsCpf1 (more than 30-fold)-induced mutagenesis. Deleting *SAE2* or overexpressing *MRE11*-H125N substantially diversified CRISPR/SpCas9 or AsCpf1-induced mutation 2–3-fold at *URA3* locus, and 3–5-fold at *ADE2* locus. Thus, the error-prone DNA repair protein was employed to develop a novel mutagenic genome editing (mGE) strategy, which can increase the mutation numbers and effectively improve the ethanol/glycerol ratio of Saccharomyces cerevisiae through modulating the expression of *FPS1* and *GPD1*. This study highlighted the feasibility of potentially reshaping the capability of genome editing by regulating the different DSB repair proteins and can thus expand the application of genome editing in diversifying gene expression and enhancing genome evolution.

**IMPORTANCE** Most of the published papers about nuclease-assisted genome editing focused on precision engineering in human cells. However, the topic of inducing mutagenesis via error-prone repair has often been ignored in yeast. In this study, we reported that perturbing DNA repair, especially modifications of the various DSB end resection-related proteins, could greatly improve the mutational efficiency and diversity, and thus functionally reshape the capability of the different genome editing tools without requiring an exogenous DNA template in yeast. Specifically, mutagenic genome editing (mGE) was developed based on CRISPR/AsCpf1 and *MRE11*-H125N overexpression, and used to generate promoters of different strengths more efficiently. Thus, this work provides a novel method to diversify gene expression and enhance genome evolution.

## INTRODUCTION

The diversified genomic alterations and perturbation of gene expression have been found to be relevant to every aspect of genetics, and can effectively facilitate genome evolution of species and development of novel or desired phenotypes (1). The various existing approaches, e.g., Delitto Perfetto (2), MAGE (3, 4), Tn-seq (5), and TRMR (6), can enable rapid mutagenesis, in combination with CRISPR/Cas9, CREATE (7), CRMAGE (8), CHAnGE (9), and others (10) to generate diversified genetic modifications more efficiently and extensively. However, these methods are usually based on homology-directed repair (HDR), and thus limited by the inefficient HDR in some microorganisms, particularly in the nondividing cells, and also sometimes involve undesired work to prepare exogenous templates.

“Template-free” DNA repair machinery was recently employed to introduce site-specific mutations (11, 12). In this process, the researchers examined the mutational landscape induced by CRISPR/Cas9 via non-homologous end-joining (NHEJ) and were surprised to find that DNA repair profiling revealed both nonrandom and predictable outcomes (13–15). The majority of the reproducible mutations generated by CRISPR/Cas9 were observed to be insertions of a single base (upstream of the cleavage site), and only a few short deletions or longer microhomology-mediated deletions were found. Although recent studies have confirmed that employing various nucleases (e.g., Cpf1, Cas9-D10A, and Cas9-N863A variants) to modulate the polarities of DSB structures and orientations can enable activating specific repair mechanisms and result in different mutational outcomes, the mutation types studied are still limited and need to be significantly improved for diversifying genomic mutagenesis and enhancing the capability of genome editing (13, 16, 17). On the contrary, base editings (BEs), e.g., CRISPR-X, CRISPR-BEST, and GBEs (18–20), can also enable catalyzing of the genetic base transitions (C to T and A to G) or base transversions (C-to-A in *E. coli* and C-to-G in mammalian cells) without the requirement of exogenous DNA templates, and thus open up new avenues for the microbial genome engineering (21). However, the mutation types introduced by BEs are primarily transformations, and often exclude deletions or insertions. In addition, the narrow editing window of BEs might serve as another major limitation in genome engineering.

To improve the capability of genome editing and expand its potential applications, ongoing efforts mainly focus on the optimization of guide sequence and customizable nucleases (22, 23). Recently, targeted modulation of DSB machinery or proteins to boost the capability of precise editing has shown great potential (24, 25), but its application in “error-prone” editing has often been ignored. Organisms have evolved two main mechanisms to repair DSB lesions: homologous recombination (HR) and NHEJ. NHEJ can be subdivided into the classical (c-NHEJ) and a highly error-prone NHEJ pathway, termed alternative NHEJ (a-NHEJ). Compared with homologous recombination, NHEJ plays a crucial role in producing error-prone outcomes in Saccharomyces cerevisiae. However, a number of previous studies have reported that only limited and inefficient mutations were generated due to the low activity of NHEJ in S. cerevisiae (26, 27). The DSB repair process generally involves the interplay of different proteins that can function to facilitate DSB end protection, tether, resection (initiating, limiting, and extending resection), and ligation, as well as DNA recombination and mismatch correction, etc. For instance, Ku70p forms a Ku heterodimer with Ku80p to protect DSB ends from nucleolytic degradation. *DNL4* encodes DNA ligase IV, which plays a pivotal role in nonhomologous end-joining. Inactivation of either Ku70p or Dnl4p can dramatically reduce the performance of c-NHEJ (28). The MRX (Mre11-Rad50-Xrs2) complex is involved in DSB repair by providing the structural support and anchoring the DNA damaged ends. *MRE11* deletion can result in severely defective c-NHEJ (28). In a-NHEJ, the MRX complex and the endonuclease Sae2p have been found to be engaged in restricting resection. The *MRE11*-H125N allele, which lacks nuclease function but retains the capacity to produce the MRX complex, possesses a significantly lower a-NHEJ and a higher c-NHEJ (29). Sae2p initiates 5′–3′ end resection and takes an active part in the damping of DNA damage signaling. The *SAE2-*S267E mutation may promote a-NHEJ by constitutively complementing Ser267 phosphorylation (30), while *MRE11*-H125N can effectively suppress the DNA damage sensitivity of *sae2*Δ cells by accelerating the turnover of Mre11p at DNA ends (31). *EXO1*, encoding 5′–3′ exonuclease, processes the limited resection intermediate to generate extensive resection of ssDNA for HR components (32). Fun30p is also involved in extensive resection by promoting exonuclease Exo1p-dependent resection, and *FUN30* deletion can reduce the rate of resection about 3-fold (32). Rad51p, also known as RecA, is usually used to identify homologous template DNA during the repair of DSBs, which is a critical step in HR. Inactivation of Rad51p for DNA recombination can result in the accumulation of DSBs and reduce the efficiency of HDR (33). Msh2p is a member of the MutS homologs (MSH) family of proteins that aids in mismatch repair (34). Thus, modulating these various key proteins can significantly influence the DSB repair process (35, 36). Overall, modifications of the different DSB repair pathways or proteins could serve as a promising approach to develop a novel template-free genome editing tools for diversifying genomic modifications and promoting genome evolution.

Here, we systematically investigated the ability of four commonly used genome editing tools, which can effectively generate various DSB structures (Fig. S1 in the supplemental material) (37–40). Then, we introduced the random genetic mutations without exogenous DNA template, and thereby significantly improved the efficiency for producing a diversified mutational landscape by modulating DSB end resection proteins in S. cerevisiae. Furthermore, we developed a novel mutagenic genome editing (mGE) strategy that could markedly increase DSB repair performances to potentially accelerate genome evolution and aid in screening the mutants with desired phenotypes. We also discuss the future prospects to extend efficient genome editing beyond S. cerevisiae.

## RESULTS

### Different genome editing tools prompted different but limited mutational landscapes in S. cerevisiae.

**FIG 1:**
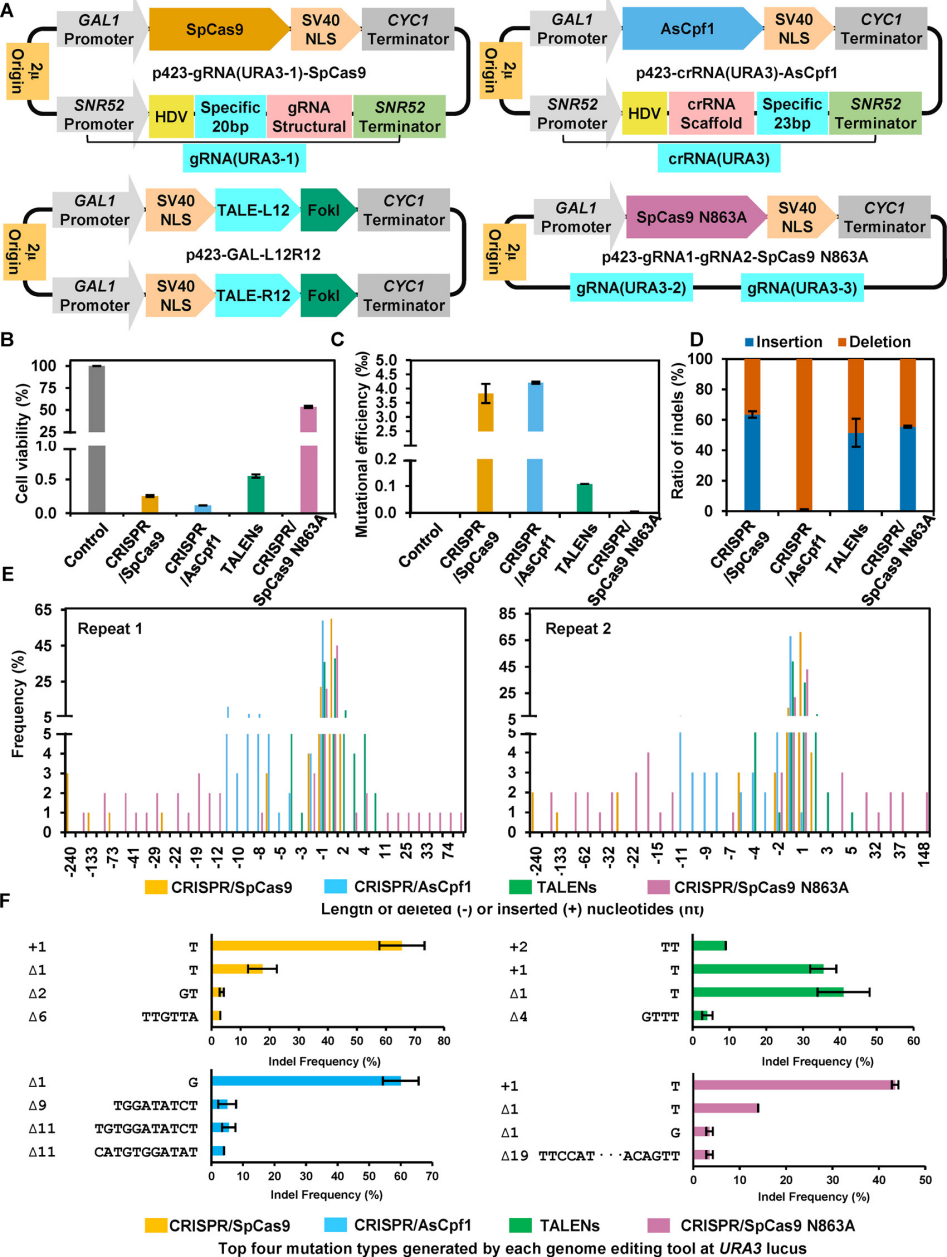
Evaluation and comparison of the mutational landscape generated by the different editing tools. (A) Diagram of the plasmids containing programmable nucleases and guide modules for the different genome editing tools. SpCas9, AsCpf1, and SpCas9 N863A were fused with C-terminal SV40 nuclear localization signal sequence and thereafter expressed under the control of *GAL1* promoter and the *CYC1* terminator. TALEN modules with 12 bp target sequences (left and right), the nuclease FokIs at C termini, and SV40 at N termini were also expressed under the control of *GAL1* promoters and the *CYC1* terminators. The gRNA containing self-cleaving hepatitis delta virus (HDV) ribozyme, 20 bp specific target sequence for *URA3* (*URA3*-1, *URA3*-2, or *URA3*-3), and the structural components were expressed under the control of snoRNA *SNR52* promoter and the terminator of yeast *SUP4*. The crRNA containing HDV ribozyme, crRNA scaffold, and 23 bp specific target sequence for *URA3* was expressed under the control of *SNR52* promoter and *SUP4* terminator. (B) The cell viability of BY4741a after different genome editing at *URA3* locus. (C) Mutational efficiency of BY4741a after the different genome editing at *URA3* locus. (D) The ratios of the deletion and insertion generated by the different genome editing tools at *URA3* locus in BY4741a. The different repair outcomes were measured by PCR amplification of the target *URA3* locus from randomly selected 100 individual colonies in SD + 5-FOA plate (Fig. S2), followed by DNA sequencing. The ratio was calculated based on the number of inserted or deleted mutations at the target *URA3* loci. (E) Frequency for the size of inserted (+) or deleted (–) nucleotides at the vicinity of the target loci generated by the different genome editing tools. (F) The top four mutational types were generated by four different editing tools. The data of (D), (E), and (F) were calculated from DNA sequencing results of above analyzed 100-individual colonies. Error bars were derived from two different biological triplicates. Control: BY4741a harboring pRS423; CRISPR/SpCas9: BY4741a harboring p423-gRNA(*URA3*-1)-SpCas9; CRISPR/AsCpf1: BY4741a harboring p423-crRNA(*URA3*)-AsCpf1; TALENs: BY4741a harboring p423-GAL-L12R12; CRISPR/SpCas9 N863A: BY4741a harboring p423-gRNA1-gRNA2-SpCas9 N863A.

To elucidate mutational landscapes introduced by the different genome editing tools without exogenous DNA template, four different editing tools with different cleavage patterns (Fig. S1) including CRISPR/SpCas9, CRISPR/AsCpf1, TALENs, and CRISPR/SpCas9 N863A were applied to mutate *URA3* gene locus in S. cerevisiae. These four programmable nucleases were thereafter expressed under the control of the *GAL1* promoter (Fig. 1A) that has been reported to be tightly repressed by glucose but strongly induced by galactose (41), thus allowing the cells to regulate genome editing by switching the various carbon sources.

We first evaluated the effects of these four genome editing tools on the cell viability and mutational efficiency (exactly, *URA3* inactivation frequency) based on the plate counting and the sequencing of the genomic modifications at the *URA3* loci (Fig. S2A) (27). Upon induction, the control strain without any editing tool exhibited cell viability close to 100%, whereas cell viability of the strains with CRISPR/SpCas9, CRISPR/AsCpf1, or TALENs significantly decreased to less than 1%, but interestingly, the cell viability of the strain with CRISPR/SpCas9 N863A was still found to be 53.44% (Fig. 1B). The lethality generated by these genome editing tools showed direct correlations with mutational efficiency, as CRISPR/AsCpf1 led to the lowest cell viability but the highest *URA3* mutational efficiency of 4.2%, whereas CRISPR/SpCas9 N863A demonstrated opposite trends with highest cell viability but the lowest mutational efficiency (Fig. 1B and C).

To characterize the detailed mutation landscapes, 100 different isolated colonies on 5′-FOA plates from each editing tool were randomly picked and their DNA regions containing *URA3* loci were amplified by PCR and subsequently sequenced. The results identified that all the mutations were located near the gRNAs or TALE binding sequences (Data Set S1). The insertion rates caused by CRISPR/SpCas9, TALENs, and CRISPR/SpCas9 N863A (63.5, 51.5, and 55.5%, respectively) were slightly higher than that of the deletion, while only deletions were detected for CRISPR/AsCpf1 (Fig. 1D). Interestingly, TALENs and CRISPR/AsCpf1 both yielded similar 5′ overhang DSBs, but the repair outcomes were markedly different (Fig. S3B, S3C, and Fig. 1D). Furthermore, the size of indels significantly differed among these four genome editing tools. CRISPR/SpCas9 induced DSBs were occasionally resolved with relatively larger deletions, such as –240 nucleotides (nt), and the size of indels induced by CRISPR/SpCas9 N863A varied in the range of –159 to +148 nt (Fig. 1E). We also found that CRISPR/SpCas9 N863A induced insertions were frequently detected within the predicted overhang, thereby suggesting 3′-dependent oligonucleotides “fill-in” (Fig. S3), likely by using the a-NHEJ pathway as previously reported (42).

Overall, only 11, 16, 8, and 30 types of insertions and/or deletions (9, 16, 9, and 23 for the repeat experiment) were observed for CRISPR/SpCas9, CRISPR/AsCpf1, TALENs, and CRISPR/SpCas9 N863A, respectively (Fig. S3 and Data Set S1). Among them, around 50% of DSBs were found to be repaired as 1-nt insertions and/or deletions (Fig. 1E and F), thus indicating low diversity of mutagenesis induced by these genome editing tools in the absence of exogenous DNA templates. Taken together, these results clearly suggested that four commonly used genome editing tools induced limited but different mutational landscapes, thus suggesting that different DSB repair machineries were potentially engaged.

### Modulating DSB repair proteins could significantly improve the mutational efficiency as well as cell viability.

We next aimed to understand the formation of the various mutational landscapes and to identify the promising targets to trigger more efficient error-prone DSB repair. Hence, we tested the mutational efficiency and cell viability by using different genome editing tools in the context of modifying the series of distinct DNA repair proteins involved in DSB end protection, tether, resection, and ligation, as well as DNA recombination and the mismatch correction (Fig. S4).

**FIG 2:**
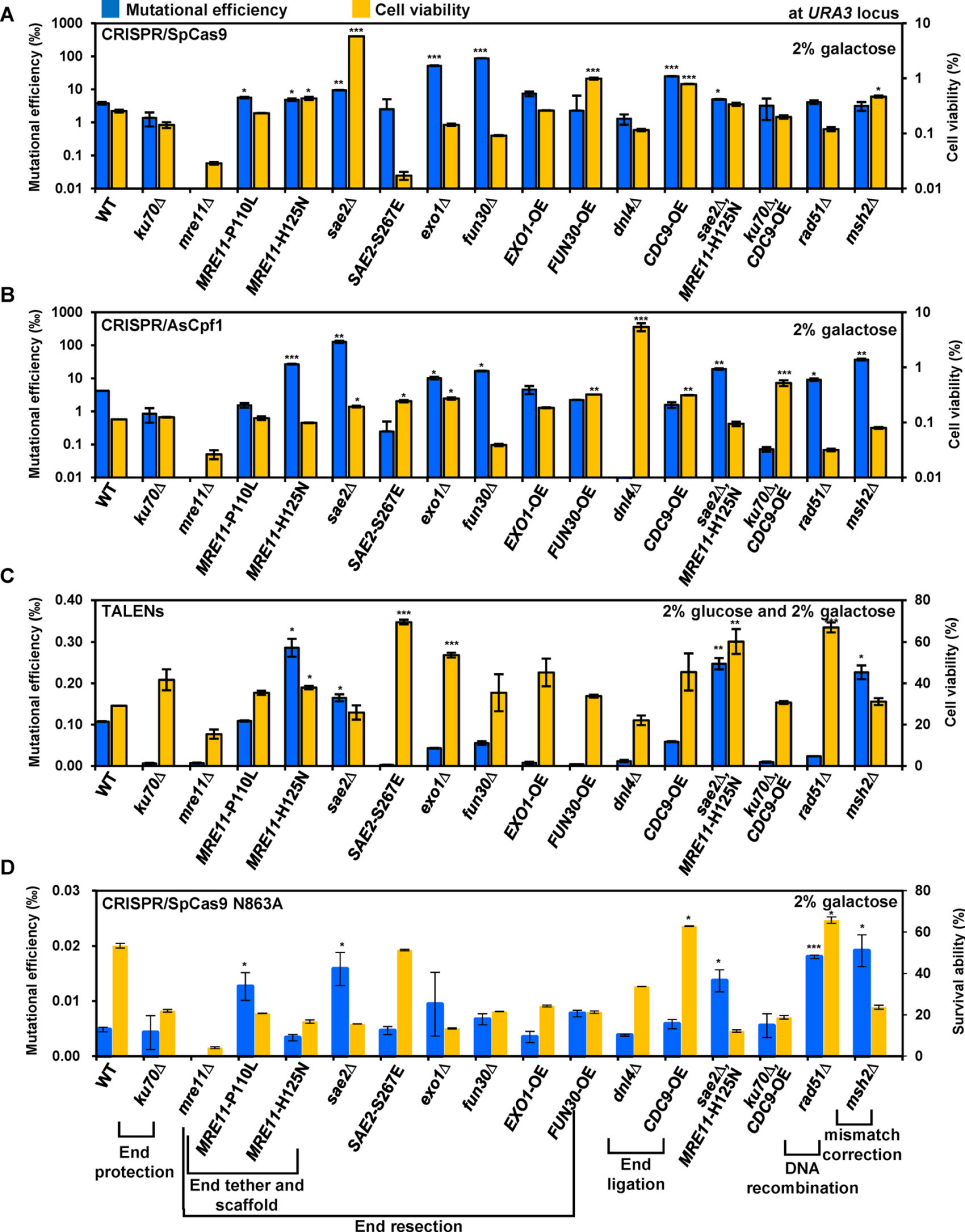
Potential effects of modulating DSB repair proteins on mutational efficiency and cell viability for editing tools. (A) CRISPR/SpCas9, (B) CRISPR/AsCpf1, (C) TALENs, and (D) CRISPR/SpCas9N863A-induced mutational efficiency and cell viability were evaluated in the various strains with DSB repair related genetic modifications via gene deletion, overexpression, or mutations. Notably, the TALENs were performed under mixed sugar conditions (2% glucose with 2% galactose). Blue column: mutational efficiency; yellow column: cell viability. All the different strains contained p423-gRNA(*URA3*-1)-SpCas9, p423-crRNA(*URA3*)-AsCpf1, p423-GAL-L12R12, and p423-gRNA1-gRNA2-SpCas9 N863A, respectively, for the different genome editing (Fig. 1A and Table S1). Statistical analysis was performed using one-way ANOVA followed by Tukey’s multiple-comparison posttest (***, *P* < 0.001; **, *P* < 0.01; *, *P* < 0.05).

The effect of CRISPR/SpCas9 at the *URA3* locus was studied first. The results indicated that single-gene deletion of *SAE2*, *EXO1*, or *FUN30* for DSB end resection as well as single gene overexpression of *CDC9* for DSB end ligation significantly increased mutational efficiency (>2-fold), and *FUN30* deletion exhibited the highest mutational efficiency (22.7-fold increase). In addition, *SAE2* deletion dramatically increased the cell viability (22.8-fold) (Fig. S5). As a result, inactivating Sae2p was observed to improve both CRISPR/SpCas9-induced mutational efficiency and cell survivability (Fig. S6). However, overexpression of *MRE11*-P110L (reduced DNA binding) or *MRE11*-H125N (nuclease deficiency) in *mre11*Δ strain only displayed a slight increase in the mutational efficiency compared to the control (Fig. 2A and Fig. S5). These results suggested that DSB end resection had the dominant effect in increasing the mutational efficiency in the presence of CRISPR/SpCas9-induced DSB repairs. The similar observation at *ADE2* locus further confirmed this conclusion (Fig. S7A). In particular, deleting *SAE2* or overexpressing *MRE11*-H125N markedly increased the mutational efficiency of CRISPR/SpCas9 by 12.8- and 19.9-fold, respectively. Furthermore, overexpression of *CDC9* for DSB end ligation augmented the mutational efficiency by 6.5-fold. Overall, it was observed that manipulating the DSB end resection related genes (deleting *SAE2*, *EXO1*, or *FUN30*, or overexpressing *MRE11*-H125N) could significantly increase the mutational efficiency of CRISPR/SpCas9-based genomic modifications.

For CRISPR/AsCpf1, single-gene deletion of *SAE2*, *EXO1*, and *FUN30* for DSB end resection, *RAD51* for homologous recombination, and *MSH2* for mismatch correction increased the mutational efficiency (>2-fold) at *URA3* locus, and deletion of *SAE2* showed the highest increased mutational efficiency (30.2-fold increase) (Fig. 2B and Fig. S5). In addition, the cell viability induced by CRISPR/AsCpf1 was found to increase from 0.11% in the wild type (WT) to 0.19% with Sae2p inactivation. The cell viability was proportional to the mutation efficiency in the *sae2* deletion mutant strain (Fig. S6). Similar to CRISPR/SpCas9, overexpression of *MRE11* alleles (*MRE11*-H125N or *MRE11*-P110L) could dramatically restore the mutational efficiency and cell viability of the *mre11* null mutant strain, whereas an overexpression of *MRE11*-H125N even increased the mutational efficiency more than 6-fold compared with that of WT. Moreover, deleting *SAE2* or *EXO1*, or overexpressing *MRE11*-H125N, increased the mutational efficiency 7.0-, 1.7-, and 4.7-fold, respectively at *ADE2* locus (Fig. S7A). In summary, these results implied the function of Mre11p with nuclease deficiency can facilitate an error-prone repair of DSBs generated by CRISPR/AsCpf1, and deleting *SAE2* for DSB end resection could be greatly beneficial to enhance the mutational efficiency of CRISPR/AsCpf1-based genomic modifications.

**FIG 3:**
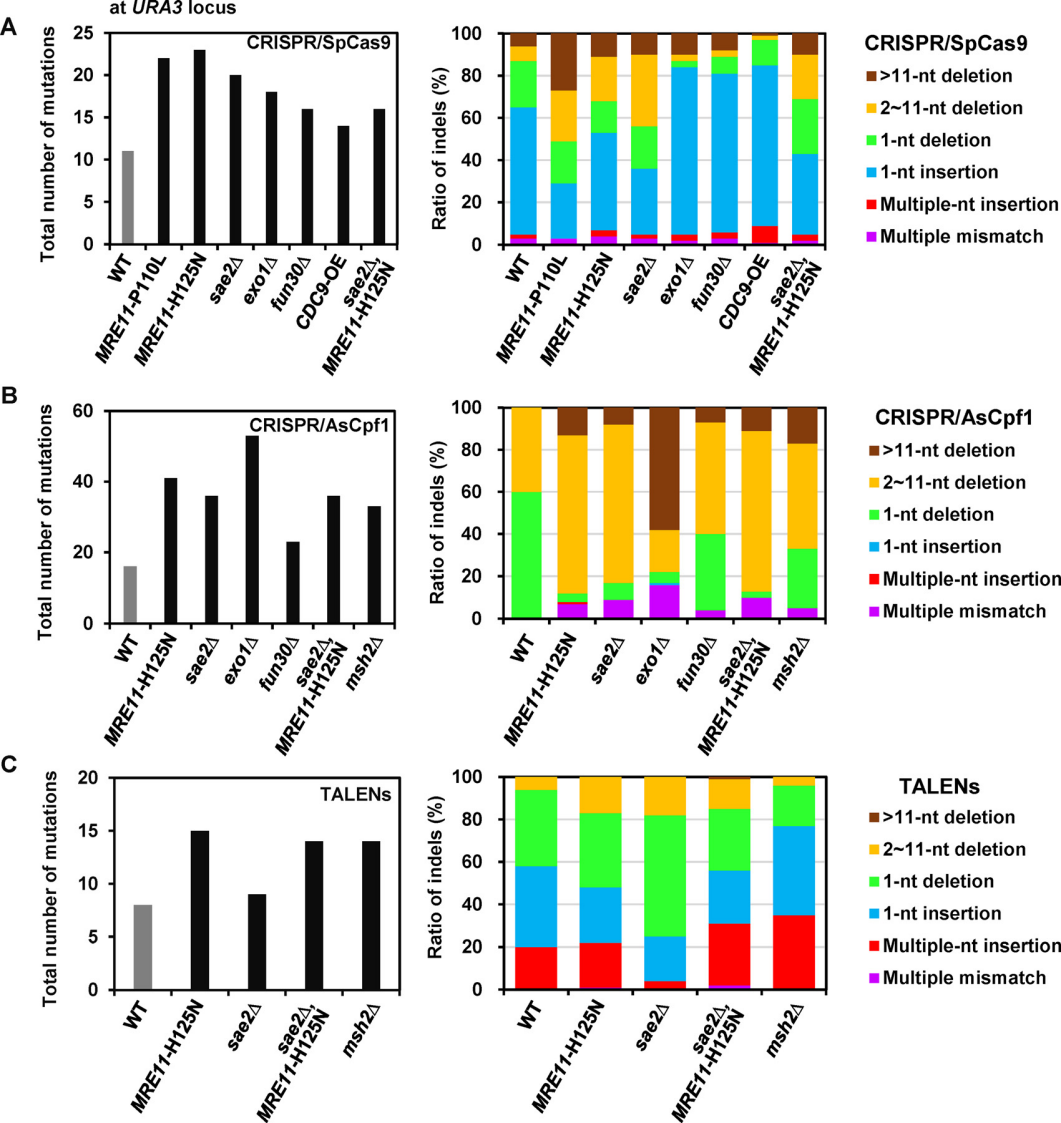
Possible effects of modulating DSB repair proteins on mutational diversity for editing tools. (A) CRISPR/SpCas9, (B) CRISPR/AsCpf1, and (C) TALENs-induced total mutational diversity were evaluated in the various strains with DSB repair-related genetic modifications via inducing gene deletion, overexpression, or mutation. The total number of mutations of the wild-type and genetically modified strains after different genome editing is shown in the left panel, and these numbers were counted by PCR amplification of the *URA3* loci from randomly selected 100-individual colonies of each strain with the different modulation and genome editing tools, followed by DNA sequencing. Correspondingly, the sequencing outcomes were further analyzed by classifying the different groups based on mutation types, including >11-nt deletion, 2–11-nt deletion, 1-nt deletion, 1-nt insertion, multiple-nt insertion, and multiple mismatches, and the ratio of each group was calculated as shown in the right panel. Multiple mismatches observed represent the mutagenesis sequence containing both the deletion and insertion. All the strains contained p423-gRNA(*URA3*-1)-SpCas9, p423-crRNA(*URA3*)-AsCpf1, p423-GAL-L12R12, and p423-gRNA1-gRNA2-SpCas9 N863A, respectively, for the different genome editing (Fig. 1A and Table S1).

The mutational efficiency of TALENs is insufficient, and only a few mutants were created, thus making it impossible to meet the criteria of the mutation detection (Fig. S8). A mixed sugar strategy (2% glucose with 2% galactose) was used to introduce adequate mutants for calculating the mutation efficiency (Fig. 2C) and characterizing the mutation numbers and types (Fig. 3C). In this “two-step” strategy, glucose is used to generate a large number of cells, and then galactose-induced nucleases are employed to introduce the DSB lesion. Both CRISPR/AsCpf1 and TALENs generated similar 5′-cohesive overhanging DSBs, but the potential effects of different DSB repair modulations on the mutational efficiency and cell viability were observed to be quite different. Unlike CRISPR/AsCpf1, attenuation of the activations of DSB end resection exhibited limited positive effects on the mutational efficiency through TALENs, but overactivated DSB end resection significantly decreased the mutational efficiency. In wild type stain, CRISPR/AsCpf1 only caused deletions, whereas TALENS induced deletions and insertions in roughly similar proportions (Fig. 1D). This result indicated that DSB end resection proteins might perform dramatically diverse roles in processing overhangs produced by CRISPR/AsCpf1 and TALENs. Especially, overexpression of *MRE11*-H125N in *mre11*Δ strain markedly relieved the negative effect of *MRE11* deletion and further increased the mutational efficiency 2.7-fold. *SAE2* deletion had almost minimal effect on the mutational efficiency while overexpression of *SAE2-*S267E in *sae2*Δ strain substantially decreased the mutational efficiency (49.1-fold). The combination of *SAE2* deletion and *MRE11*-H125N overexpression in the *mre11*Δ strain showed a 2.3-fold increase in the mutational efficiency, which might be due to the positive contribution of *MRE11*-H125N overexpression. Surprisingly, deleting or overexpressing both DSB end resection genes *EXO1* and *FUN30* decreased the mutational efficiency, but overexpression showed a more significant effect on the decreased mutational efficiency (Fig. 2C and Fig. S5).

For CRISPR/SpCas9 N863A, overexpressing *MRE11*-P110L instead of *MRE11*-H125N in the *mre11*Δ strain markedly rescued the destroyed mutagenic repair of DSBs and significantly increased the mutational efficiency. Similar to CRISPR/SpCas9, the deletion of *SAE2* or *EXO1* genes significantly increased the mutational efficiency. Additionally, similar to CRISPR/AsCpf1, single-gene deletion of *RAD51* and *MSH2* significantly increased the mutational efficiency (≥2.0-fold). Moreover, with respect to the cell viability, modulations of DSB repair proteins exhibited negative effects to different extents except for *SAE2-*S267E overexpression, *CDC9* overexpression, and *RAD51* deletion (Fig. 2D and Fig. S5). These results suggested that CRISPR/SpCas9 N863A-induced DSBs might be predominantly subjected to the precise repair mechanism rather than the mutagenic repair.

Overall, selective modulation of the repair proteins, especially in DSB end resection process, could improve the mutational efficiency of the various genomic modifications, but the extents of improvement varied depending on the different genome editing tools. To be specific, the CRISPR/SpCas9-based mutational efficiency was increased from 3.8% to 86.9% (22.9-fold) when deleting *FUN30* for DSB end resection (Fig. 2A). The CRISPR/AsCpf1-based mutational efficiency was also enhanced from 4.2% to 126.8% (30.2-fold) when deleting *SAE2* for DSB end resection (Fig. 2B). The TALENs-based mutational efficiency was augmented from 0.11% to 0.29% (2.6-fold) when expressing *MRE11*-H125N related to the resection initiation (Fig. 2C). The CRISPR/SpCas9 N863A-based mutational efficiency was increased from 0.0048% to 0.0191% (4.0-fold) upon deletion of the *MSH2* gene for the mismatch correction (Fig. 2D). Thus, the results clearly suggested that modulating DSB end resection proteins could significantly increase the mutational efficiency of CRISPR/SpCas9 and CRISPR/AsCpf1. Furthermore, overexpression of *CDC9* and deletion of *RAD51* or *MSH*2 also significantly improved CRISPR/SpCas9 or CRISPR/AsCpf1 mutational efficiency (Fig. 2 and Fig. S5). Whether these beneficial DSB repair proteins can further contribute to diversify the mutational landscape needs further investigation.

### Modulating DSB end resection-related proteins were found to significantly diversify the genomic alterations.

Mutational diversity creation is central to facilitating the genome evolution and developing novel or desirable phenotypes (43, 44). The above results confirmed that modulating the different DSB repair proteins could significantly improve the mutational efficiency and cell viability. Here, we further characterized the effects of modulating some key DSB repair proteins on the mutational diversity of the genomic modifications introduced by CRISPR/SpCas9, CRISPR/AsCpf1, and TALENs genome editing tools. CRISPR/SpCas9 N863A-induced diversity could not be further analyzed due to the significantly low mutational efficiency (Fig. 2D and Fig. S5). We sequenced PCR amplicons of the targeted *URA3* loci from 100 randomly isolated 5-FOA-resistant colonies for each modification with enhanced mutational efficiency. In addition, we also performed amplicon sequencing of the targeted loci at *URA3* and *ADE2* for each modification of DSB end resection proteins. Based on the sequencing results, the total number of mutations and the ratio of the mutation types resulting from the genome editing were analyzed and compared (Data Sets S1 and S2).

For CRISPR/SpCas9, the result of clone sequencing indicated that the single-gene deletion of *SAE2*, *EXO1*, or *FUN30* as well as overexpressing *CDC9*, *MRE11*-H125N, or *MRE11*-P110L markedly increased the mutational diversity at *URA3* locus (Fig. 3A). Thus, modulating the proteins for DSB end tether and resection initiation, including overexpression of *MRE11*-H125N or *MRE11*-P110L in the *mre11*Δ strain, exhibited an approximately 2-fold increase in the number of mutations in contrast to that of WT, and *MRE11*-H125N overexpression showed the highest diversity. CRISPR/SpCas9 induced 11 different mutations in WT, 60% of which were 1-nt insertion (Fig. 3A, right panel). However, compared with WT, an overexpression of *MRE11*-P110L with reduced DNA binding or *MRE11*-H125N with nuclease deficiency in *mre11*Δ strain as well the deletion of *SAE2* for DSB end resection substantially decreased the ratio of 1-nt insertion while increasing the ratio of 2–11 and >11-nt deletion. On the contrary, the deletion of *EXO1* and *FUN30* genes for DSB extensive resection increased the ratio of 1-nt insertion (75 and 79%, respectively). These results implied that *MRE11*, *SAE2*, *EXO1*, and *FUN30* genes exhibited different roles in DSB end resection, and thus led to the great differences in the repair outcomes and mutational landscapes. The combination of deleting *SAE2* and overexpressing *MRE11*-H125N in the *mre11*Δ strain showed no synergetic effects of further increasing the mutational diversity, although the total number of the mutation increased markedly compared to WT. In addition, overexpression of the *CDC9* gene for DNA ligation in a-NHEJ also apparently increased the ratio of 1-nt insertion (76%) compared to WT. We also employed amplicon sequencing to study the mutational diversity at another locus (*ADE2*) and found that modulating DSB end resection proteins could significantly increase the total number of mutations 2–5-fold (Data Set S2 and Fig. S7B). Overall, the perturbation of the different DSB repair genes related to DSB ends resection, including *MRE11*, *SAE2*, *EXO1*, and *FUN30* genes, significantly enhanced the mutational diversity of CRISPR/SpCas9-mediated genomic modifications.

For CRISPR/AsCpf1, the result of the clone sequencing clearly indicated that deleting *SAE2*, *FUN30*, *EXO1*, or *MSH2* genes and overexpressing *MRE11*-H125N increased the mutational diversity at the *URA3* locus (Fig. 3B). Hence, modulating the different proteins involved in DSB end tether and resection initiation as well as in mismatch correction, including overexpression of *MRE11*-H125N in the *mre11*Δ strain, and *SAE2*, *EXO1*, or *MSH2* deletion, exhibited about a 2-fold increase in the number of mutations in contrast to WT. Surprisingly, the deletion of *EXO1* had 53 mutations, much greater than 16 mutations of WT. Among the 16 different mutations found in WT, 60% and 40% were 1- and 2–11-nt deletions, respectively (Fig. 3B, right panel). Compared with WT, modulating the various DSB repair proteins decreased the ratio of 1-nt deletion, but increased the ratio of 2–11/>11-nt deletion and remarkably led to the formation of other mutation groups that included both multiple mismatches and multiple-nt insertion. *MRE11*-H125N overexpression in the *mre11*Δ strain or *SAE2* deletion substantially increased the ratio of 2–11-nt deletion (75%) higher than those of *EXO1* and *FUN30* deletion. These results further implied that Mre11p, Sae2p, Exo1p, and Fun30p employed for the different DSB end resection resulted in the different repair outcomes and mutational landscapes. *EXO1* deletion greatly increased the ratio of >11-nt deletion (58%) and produced a relatively high ratio of the multiple mismatches (16%). This suggested that other extensive resection proteins, for example, *SGS1* could also possibly affect CRISPR/AsCpf1-induced repair outcomes. Furthermore, the ratio of 2–11-nt deletion resulting from *EXO1* deletion decreased 2-fold, and the ratio of 1-nt deletion, which was dominant in WT, was markedly decreased to 5%. The deletion of *MSH2* for mismatch correction resulted in 17% >11-nt deletion and 5% multiple mismatches, while the ratio of 1-nt deletion decreased 2.1-fold. Additionally, the combination of *SAE2* deletion and *MRE11*-H125N overexpression in the *mre11*Δ strain did not further increase the total number of mutations or present different ratios of indels compared to either of single modulation, thereby indicating no synergistic effects on the mutational diversity. The amplicon sequencing was next performed to reveal the mutational diversity generated by CRISPR/AsCpf1, and it showed that deleting *SAE2* and *EXO1*, and overexpressing *MRE11*-H125N, significantly increased the total number of mutations 3-, 2-, and 6-fold at the *ADE2* target (Data Set S2 and Fig. S7B). Overall, the perturbation of the different DSB repair proteins related to DSB ends resection, including Mre11p, Sae2p, Exo1p, and Fun30p, significantly enhanced the mutational diversity of CRISPR/AsCpf1-mediated genomic modifications.

For TALENs, deleting *SAE2* or *MSH2* as well as overexpressing *MRE11*-H125N was found to markedly increase the mutational diversity (Fig. 3C). Compared with CRISPR/SpCas9 and CRISPR/AsCpf1, the total number of mutations introduced by TALENs only exhibited 8 in the WT strain. Moreover, overexpressing nuclease-defective *MRE11*-H125N in the *mre11Δ* strain improved the mutations to 15; meanwhile, the ratio of 2–11-nt deletions increased 2.8-fold. Additionally, deleting *SAE2* for DSB end resection did not substantially increase the total number of mutations compared with WT strain, but the ratios of 1-nt and 2–11-nt deletions were increased by 2.8- and 1.6-fold, respectively. The increased ratio of 2–11-nt deletions resulted from perturbing *MRE11* or *SAE2*, which further confirmed the competing relationship between *MRE11*/*SAE2* and *EXO1*/*FUN30* for the different repair outcomes and mutational landscapes. Single gene deletion of *MSH2* substantially increased the number of mutations to 14 and resulted in a 1.9-fold decrease for the ratio of 1-nt deletion as well as a 1.8-fold increase for the ratio of multiple nucleotide insertion. In addition, the combination of *SAE2* deletion and *MRE11*-H125N overexpression in the *mre11*Δ strain also did not significantly increase the number of mutations and showed a similar pattern of indels to single *MRE11*-H125N overexpression rather than the single-gene deletion of *SAE2*.

In summary, modulating DSB repair, especially DSB end resection proteins, not only increased the total number of the targeted mutations but also significantly modulated the distribution of the various mutation types. The dominant repair outcomes of 1-nt insertion for CRISPR/SpCas9 or 1-nt deletion for CRISPR/AsCpf1 (Fig. 1E) in WT strain could be distinguished by the multiple-nucleotide insertion for CRISPR/SpCas9 or multiple-nucleotide deletion for CRISPR/AsCpf1 in DSB repair mutated strains at *URA3* locus (Fig. 3). Based on the increased mutational diversity and efficiency, both of which resulted from modulating the various important DSB repair proteins, the cost-effective genome editing tools could be potentially developed to effectively target the mutational landscapes for diversifying cellular phenotypes and accelerating genome evolution.

### A mutagenic genome editing (mGE) strategy was developed to diversify the gene expression.

**FIG 4:**
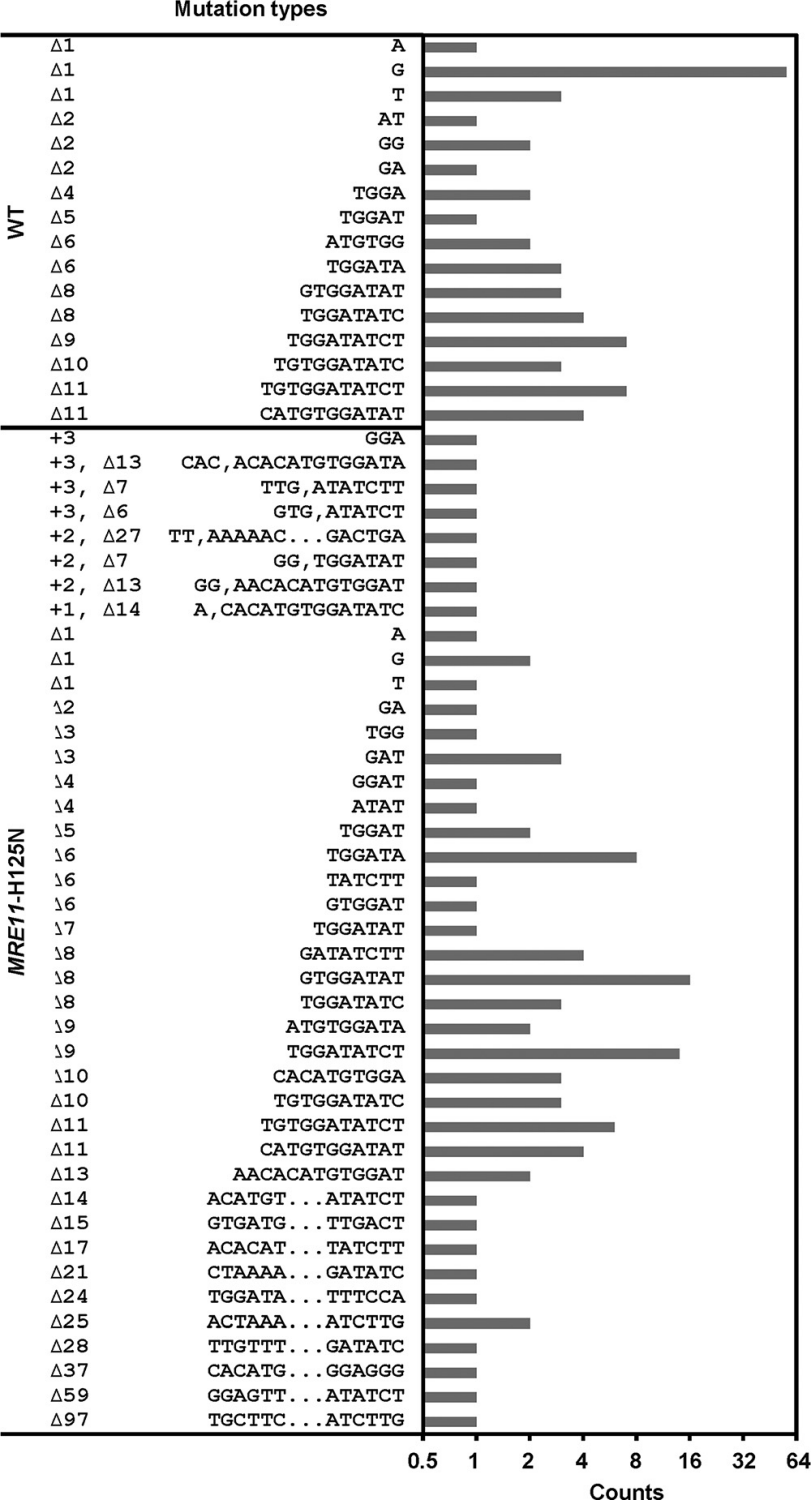
Comparison of the mutational landscapes of CRISPR/AsCpf1 genome editing with/without *MRE11*-H125N. The mutational landscapes were measured based on DNA sequencing results of *URA3* loci from each 100 randomly selected 5-FOA-resistant mutants (edited mutants), as described in Fig. S2. WT: S. cerevisiae BY4741a harboring p423-crRNA(*URA3*)-AsCpf1; *MRE11*-H125N mutant strain: S. cerevisiae BY4741a *mre11*Δ harboring p*MRE11*-H125N and p423-crRNA(*URA3*)-AsCpf1 (Table S1 and Data Set S3).

**FIG 5:**
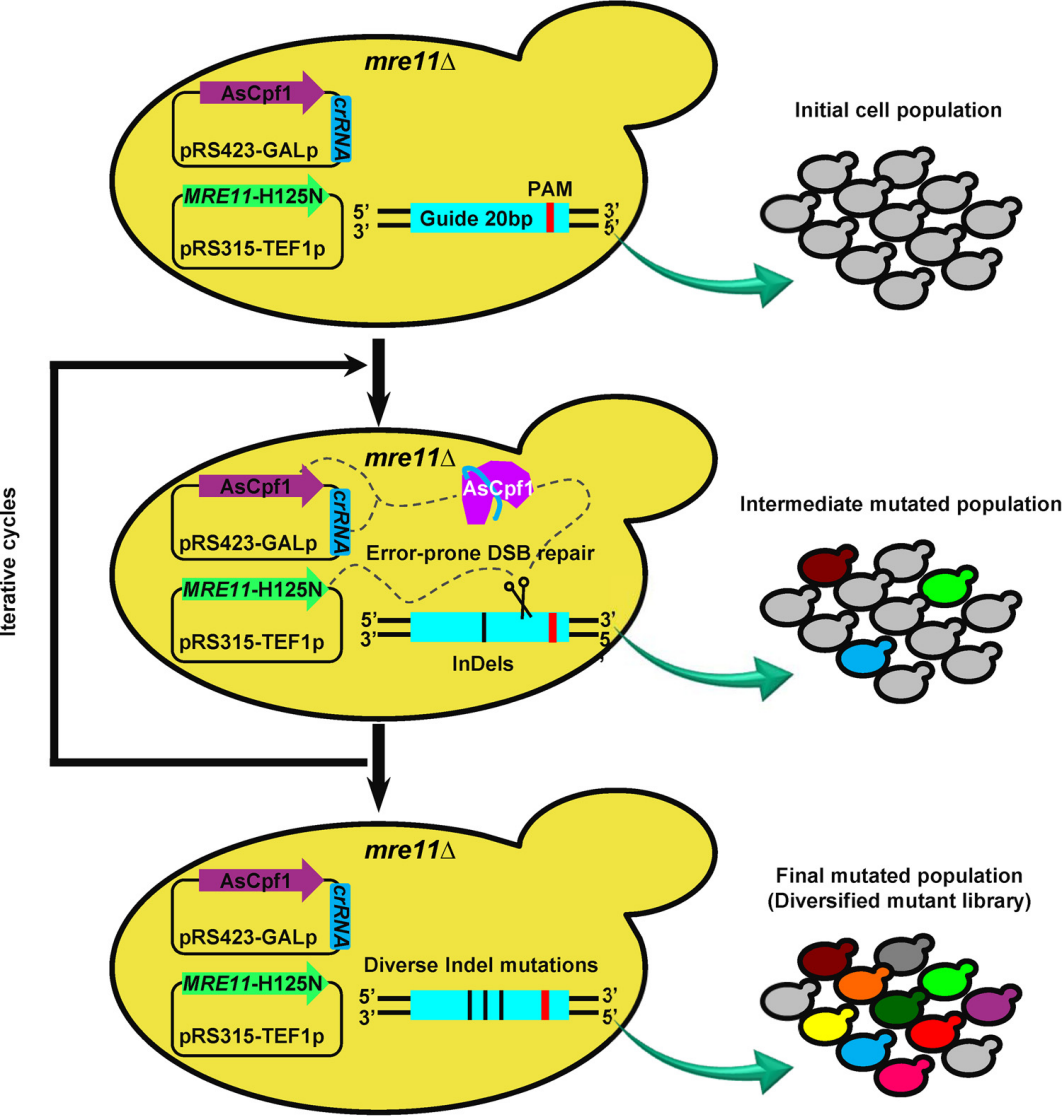
A schematic diagram of the mutagenic genome editing (mGE) strategy for improving the different cellular phenotypes. The mGE tool was developed under the cooperation between *MRE11*-H125N overexpression and CRISPR/AsCpf1. The initial *mre11*Δ yeast cell used was harboring p*MRE11*-H125N and p423-crRNA(X)-AsCpf1 (X: the target gene). In this study, *eGFP* and *FPS1* were selected as the examples and first grown in SD media containing 20 g/L glucose and the auxotrophic compounds for 12 h, and then were diluted into the SD media containing 20 g/L galactose and the auxotrophic compounds for 24 h to initiate editing. The cell population was then iteratively edited to generate the final mutated population for screening the desired phenotypes. If necessary, other genetic modification of important DNA repair proteins could also be selected to potentially replace *MRE11*-H125N overexpression for building a similar mGE strategy in combination with other genome editing tools. For example, *MRE11*-H125N overexpression could be replaced by *SAE2*, *EXO1*, *FUN30*, or *MSH2* deletion as well as *CDC9* overexpression, while CRISPR/AsCpf1 could be effectively replaced by CRISPR/SpCas9 or TALENs as well as other suitable genome editing tools.

The aforementioned results have clearly demonstrated that modulating the various DSB repair proteins, especially the proteins related to DSB end resection, could significantly impact both the mutational efficiency and diversity (Fig. 2 and 3). Hence, one of the modulations, *MRE11*-H125N overexpression, was chosen as an example to explore the possibility of modulating the capability of the genome editing and expanding their possible applications. *MRE11*-H125N overexpression could increase both the mutational efficiency and diversity, and generate a relatively uniform mutational landscape at *URA3* locus with CRISPR/AsCpf1 (Fig. 4). Thus, a mutagenic genome editing (mGE) tool was developed based on CRISPR/AsCpf1 and *MRE11*-H125N overexpression, which aimed to introduce more diverse genomic alterations and accelerate the genome evolution for improving the different cellular phenotypes (Fig. 5). In addition, we confirmed that three rounds of iteratively edited via CRISPR/SpCas9 or CRISPR/AsCpf1, but TALENs, significantly increased the mutational efficiency up to 53.85% and 56.48%, separately (Fig. S9). Thus, the *mre11*Δ yeast cell with *MRE11*-H125N overexpression and CRISPR/AsCpf1 was iteratively edited to accumulate the diversified mutations on the target locus, and then cell population after iterative editing was used to further confirm the genome evolution and screen the desired phenotypes.

**FIG 6:**
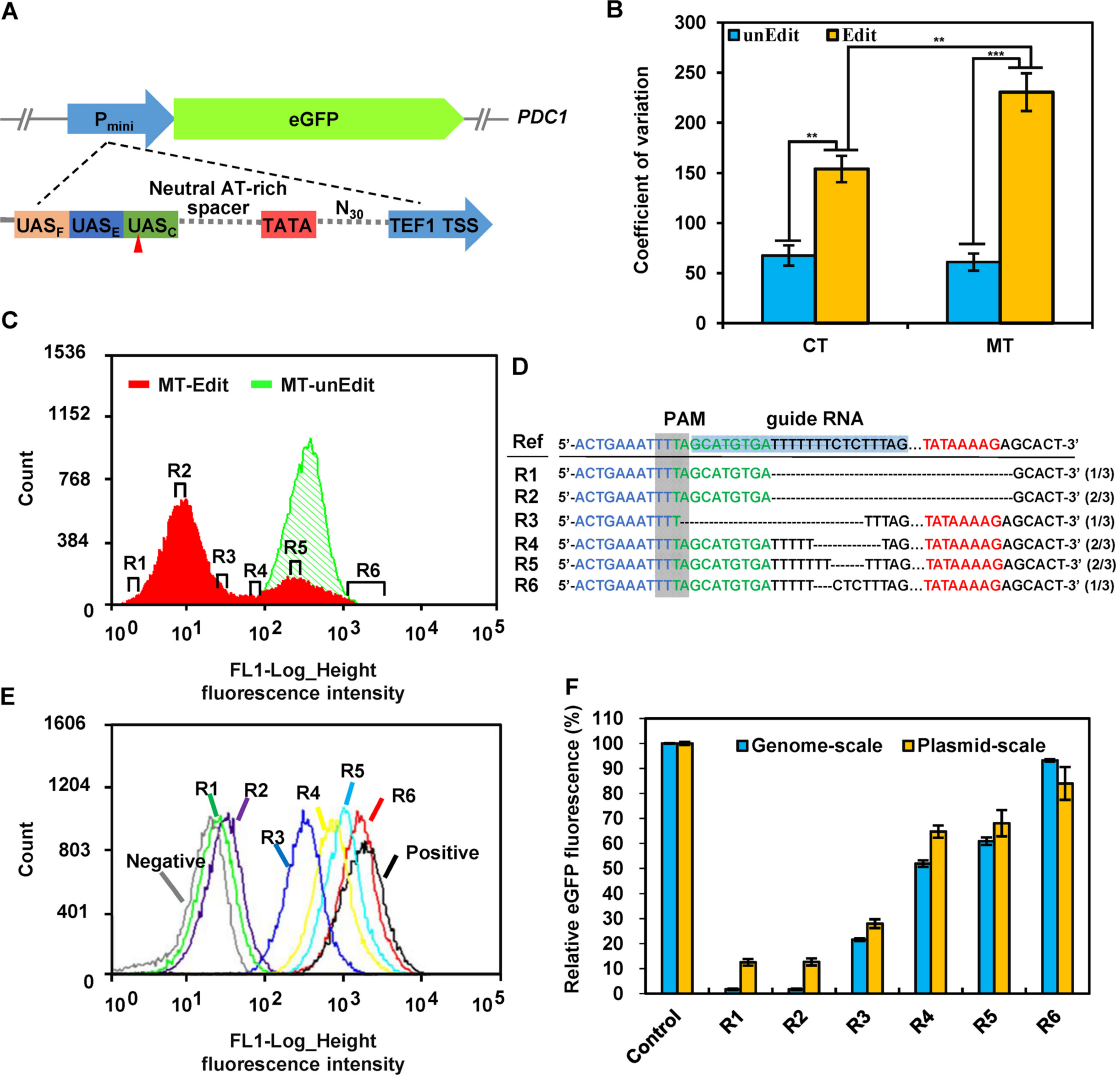
Mutational diversity of fluorescence expression resulted from the synthetic promoter editing carried out via mGE. (A) An enhanced GFP reporter driven by the synthetic minimal promoter *Pmini* was integrated to the *PDC1* locus of S. cerevisiae BY4741a, and then the mGE tools were introduced to generate *pdc1::eGFP* (CT) and *MRE11*-H125N, *pdc1::eGFP* (MT), respectively. The schematic diagram of the promoter architecture is illustrated at the bottom and the detailed sequencing information is shown in Fig. S10. (B) The distribution of the fluorescence was examined by flow cytometry, and then fluorescence diversity was characterized as coefficient variation (CV). Three random mutants from six different phenotypic diversity ranges were thereafter sorted out from iterative mGE-based edited populations (MT-Edit, R1–R6) (C) and analyzed by DNA sequencing to align their genetic variations (D). R1 and R2 appeared to have the same mutated sequences. (E) Flow cytometry histograms of the six mutants (R1–R6) and the two controls. Gray histograms represent the negative controls (no GFP expression), and black histograms represent positive controls (unedited GFP expression). The histograms of the six mutants are portrayed with green, purple, blue, yellow, sapphirine, and red, respectively. (F) The relative eGFP expression profile of the six mutants with mutated synthetic promoters at the genomes or plasmids. For genome scale, the originally sorted mutants of R1–R6 and the strain BY4741a *mre11*Δ, *pdc1::eGFP* (control) were effectively used to measure the fluorescences of eGFP expression. The fluorescence of BY4741a *mre11*Δ, *pdc1::eGFP* was first normalized to 100% and others were calculated as the values related to that of BY4741a *mre11*Δ, *pdc1::eGFP*. For the plasmid scale, S. cerevisiae BY4741a harboring the plasmid p315-Pmini(Original)-GFP, p315-Pmini(R1)-GFP, p315-Pmini(R2)-GFP, p315-Pmini(R3)-GFP, p315-Pmini(R4)-GFP, p315-Pmini(R5)-GFP, or p315-Pmini(R6)-GFP) with the enhanced GFP reporters driven by original (control) or mutated (R1–R6) synthetic minimal promoters, respectively, was used to measure the fluorescence of eGFP expression. The fluorescence of S. cerevisiae BY4741a with p315-Pmini(Original)-GFP was normalized to 100%, and others were then calculated as the values related to that of S. cerevisiae BY4741a with p315-Pmini(Original)-GFP. The error bars were derived from three different biological triplicates.

To verify the mGE strategy, the fluorescence diversity of *eGFP* (enhanced green fluorescent protein) was used to analyze the possible editing effect on promoter strengths. A synthetic promoter *Pmini* was previously reported to act as a strong promoter, whose strength was only slightly weaker than the strongest constitutive *GPD* promoter (*TDH3*) (45) (Fig. S10). The promoter *Pmini* was integrated into the *PDC1* locus of S. cerevisiae with *eGFP* and then applied to generate a diversified mutant library (Fig. 6A). After three iterative cycles of mGE, the fluorescence diversity of the yeast cell population was thereafter evaluated via flow cytometry, and indicated by coefficient variation (CV). It was found that even without CRISPR/AsCpf1 editing (AsCpf1 was not induced), cell populations of MT-unEdit and CT-unEdit showed similar fluorescence diversity (Fig. 6B). However, once AsCpf1 was induced, the various cell populations of MT-Edit and CT-Edit both exhibited significant fluorescence diversity with >2-fold increase from those of MT-unEdit and CT-unEdit, respectively. Here, MT refers to the *mre11*Δ with the *MRE11*-H125N overexpression plasmid. The wild-type strain (CT) was employed as a control for the MT in this study. In addition to the wild type, another control with Mre11p inactivation could be used in this study. However, it would be difficult to carry out this control because of the low growth ability and survivability of *mre11* deletion strains (Fig. 2B). Moreover, MT-Edit exhibited 1.5-fold higher fluorescence diversity than that of CT-Edit (Fig. 6B). These results suggested that the mGE strategy might contribute to effectively diversify the expression of *eGFP* to a greater extent. To confirm that various phenotypes observed were due to the mutated synthetic promoters rather than transient effects of CRISPR/AsCpf1, three different random mutants from six ranges with varied fluorescence values were sorted out from the MT-Edit population for further characterization (Fig. 6C). Thereafter, PCR amplicons harboring the target loci from these selected mutants were sequenced to reveal their various genetic variations.

Moreover, DNA sequencing results indicated that the target locus of the synthetic promoter had been edited by mGE to generate diverse indels (Fig. 6D), and no mutations occurred in the *eGFP* coding regions. By contrast, no genomic mutation was detected in the control populations (Fig. S11). The *eGFP* expression of these six mutants presented the different phenotypes with respect to their fluorescence intensities (Fig. 6D and E) and relative eGFP fluorescences (Fig. 6F, Genome-scale). Notably, R1 and R2 mutants showed the same mutational landscape but only a slight difference in the fluorescence signal. This might be due to the fluorescence background variance noted in cells. Thereafter, the mutated synthetic promoters from six mutants as well as the original synthetic promoter *Pmini* were further constructed in the new recombinant plasmids (Table S1) to test the diverse expression of *eGFP*. S. cerevisiae BY4741a harboring the recombinant plasmids (Fig. 6F, Plasmid-scale) had similar relative fluorescence to those six mutants harboring genomic mutations (Fig. 6F, Genome-scale). As expected, there were no significant differences in the fluorescence expression levels of R1 and R2 in the genome and the plasmid levels (Fig. 6F). In summary, this implied that the mGE could possibly diversify the expression of *eGFP* more easily by generating an effective and diversified site-specific genomic alteration.

### The mGE was successfully applied to diversify gene expression for altering glycerol and ethanol production.

**FIG 7:**
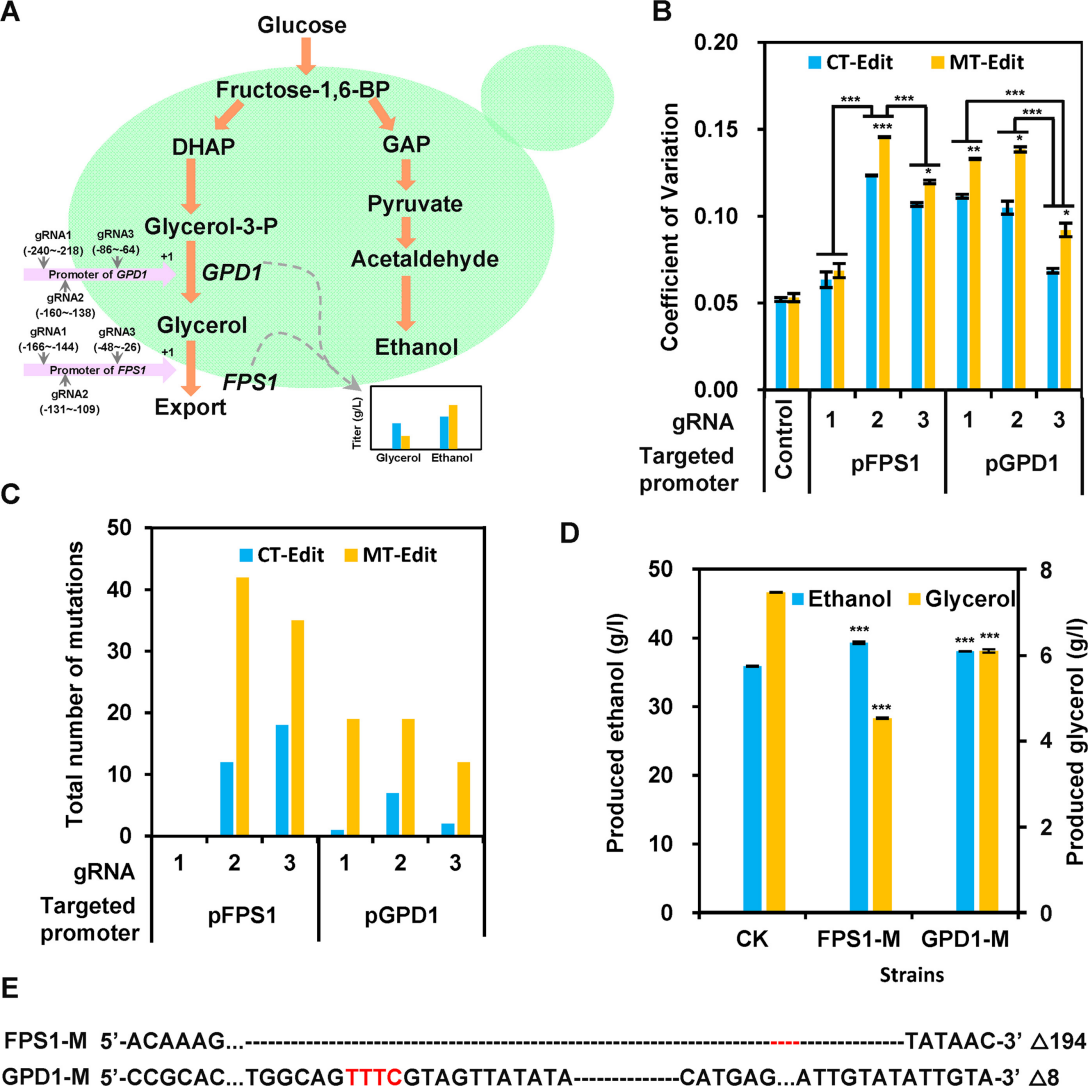
Diversification of the gene expression of *FPS1* and *GPD1* for regulating glycerol and ethanol production in yeast via mGE. (A) To alter the glycerol pathway, three different guides (gRNA1, gRNA2, and gRNA3) were designed at the native promoter of *FPS1* and *GPD1* separately. (B) The distribution of glycerol productivity of MT-Edit and CT-Edit with the different guides of *FPS1* and *GPD1* was examined in MT-Edit and CT-Edit populations (Data Set S4). BY4741 or *MRE11*-H125N mutant strain harboring blank plasmid pRS423 was then used as the control. The diversity of glycerol productivity was characterized as coefficient variation (CV). (C) The statistical analysis of the mutation types of MT-Edit and CT-Edit with the different guides of *FPS1* and *GPD1*. After three rounds of editing via mGE, the genomic DNA of each mutant library was next obtained and then used for amplifying the target locus to characterize the mutation types via amplicon sequencing (Novogene, Tianjin, China). (D) Glycerol and ethanol production profile of the two mutants with mutated pFPS1 or pGPD1 promoters. The mutants were sorted out from mGE-based edited populations (MT-Edit/gRNA2). Fermentation capacities were later evaluated at 30°C in 100-mL Erlenmeyer flasks containing 50 mL SD medium with 100 g/L glucose at 220 rpm. In panels B and D, data represent the mean and standard error of duplicate cultures under each condition. Statistical analysis was performed using one-way ANOVA followed by Tukey’s multiple-comparison posttest (***, *P* < 0.001; **, *P* < 0.01; *, *P* < 0.05). In addition, statistical analysis in B was performed using two-way ANOVA (with the strains and gNRA as the factors) followed by Tukey’s multiple-comparison posttest for selecting the best gRNA for multiplex editing. (E) The mutational landscape of the two different mutants of FPS1-M and GPD1-M.

The mGE strategy holds enormous promise to efficiently engineer S. cerevisiae by generating a diversified gene expression library. To confirm its popularity, we tested the formation of diverse expression of *FPS1* and *GPD1* genes for regulating the production of glycerol via mGE. Glycerol is one of the main by-products in bioethanol fermentation and might account for up to 5% of the substrate carbon; thus, the abolishment or at least a substantial reduction may lead to a significant increase in ethanol production (46). The native promoter of *FPS1* and *GPD1* in the S. cerevisiae genome was applied to generate a diversified mutant library via mGE. Three distinct guides (gRNA1, gRNA2, and gRNA3) were thereafter designed to target the different sites of these two native promoters (Fig. 7A).

After three iterative cycles of mGE, the glycerol production diversity of each 100 randomly selected mutant from the different yeast cell populations with different guides was analyzed via fermentation assays (Fig. S12), and indicated by CV. BY4741 and derived *MRE11*-H125N containing blank plasmids pRS315 and pRS423 without nucleases modules were used as the controls to evaluate the background of population diversity. As shown in Fig. 7B, the control strains displayed minimal phenotypic diversity, while the performance of the populations treated with mGE was observed to be more diversified. Moreover, compared with the strain without *MRE11*-H125N overexpression (CT-Edit), cell populations of the strain with *MRE11*-H125N overexpression (MT-Edit) showed a significantly increased glycerol production diversity (higher CV) after CRISPR/AsCpf1 editing (Fig. 7B).

Furthermore, compared with the control strain, MT-Edit populations of pFPS1-gRNA2 targeting to *FPS1* promoter with gRNA2, and pGPD1-gRNA2 targeting to *GPD1* promoter with gRNA2, were noted to exhibit more significant diversity with more than 1.75- and 1.60-fold increase, respectively (Fig. 7B). In addition, both CT-Edit and MT-Edit populations of pFPS1-gRNA1 targeting to *FPS1* promoter with gRNA1 showed similar diversity with the control strains, thereby clearly indicating that mGE may be ineffective at this targeted locus. To confirm that these varied phenotypes were generated due to the mutated promoters rather than transient effects of CRISPR/AsCpf1, the genomic variants of diverse populations treated with mGE were analyzed by amplicon sequencing. The result indicated that the *MRE11*-H125N mutant strain (MT-Edit) produced more abundant genetic variations at the target site than the control strain (CT-Edit). This was found to be consistent with the *MRE11*-H125N mutant strain (MT-Edit) presenting the increased glycerol production diversity (Fig. 7C and Data Set S2). Meanwhile, the results of amplicon sequencing further confirmed that modulating the DSB end resection protein (Mre11p) can enable distinct genomic alterations at five different loci (gRNA2 and gRNA3 of pFPS1, gRNA1, gRNA2 and gRNA3 of pGPD1). Unsurprisingly, no mutation was detected at the gRNA-1 locus of pFPS1, thus indicating that the gRNA-1 might have no significant activity, which was consistent with its phenotype of unaltered glycerol production compared with the unedited original strain (Fig. 7B and Fig. S12).

Two different mutants (FPS1-M and GPD1-M) with mutated *FPS1* or *GPD1* promoters showing the lowest glycerol productivity were sorted out from mGE-based edited populations (MT-Edit/gRNA2) and thereby evaluated for the capacity to promote glycerol formation and ethanol production. It was found that compared with the control strain without editing (*MRE11*-H125N mutant strain with natural *FPS1* or *GPD1* promoter), the relative glycerol productivities of these mutants (FPS1-M and GPD1-M) decreased to 39.3%, and 18.3%, respectively (Fig. 7D, and Fig. S13). Correspondingly, the ethanol production of these mutant strains displayed a 9.5% and 6.1% increase, respectively (Fig. 7D and Fig. S13). Thus, the mGE toolkit designed in this study could successfully improve ethanol production by inducing diversified genetic modifications on the promoters of *FPS1* and *GPD1* genes.

Overall, this study indicated that the mutagenic genome editing based on the modulation of the DSB repair protein could indeed improve phenotype diversity via more effectively perturbing the expression of the target genes and thus has an enormous potential to accelerate the genome evolution for obtaining the desired phenotypes.

## DISCUSSION

Interplay between genome editing-induced DNA lesions and DNA repair has been previously investigated. However, the detailed impact of DNA repair on the mutational outcomes and potential applications are still unknown. With wild-type DSB repair machinery, CRISPR/Cas9 induced-DSB lesion can result in small indels in the absence of exogenous DNA template, and this observation was consistent with a recent report (16). Using other editing tools, we have also observed an obvious shift in mutational efficiency and landscape as well as in cell viability (Fig. 1). CRISPR/AsCpf1-induced DSB lesion resulted in a 5′ 2–4-nt overhang and can predominantly generate deletion events. CRISPR/Cas9 N863A-induced 3′ overhangs led to the longer insertion events, but the mutational efficiency was significantly lower than that of CRISPR/Cas9 or CRISPR/AsCpf1. TALENs produced a 4-nt 5′ overhang and also exhibited very low mutational efficiency and cell viability, especially under the conditions of overexpressing TALENs (Fig. S8). These results clearly indicated that different DSBs induced by the different genome editing tools might recruit diverse DSB repair proteins. This study demonstrated that several DSB repair proteins, especially those related to end resection of DSBs, could greatly affect the repair outcomes of DSBs and can functionally introduce more diverse mutations.

Nonfunctional *URA3* mutants selected by 5-FOA were utilized to assess mutational efficiency as previously described (27). Interestingly, similar methods were also used in the previous studies to characterize the NHEJ-based mutational efficiency (26, 47). In this study, the mutational efficiency was calculated as a percentage only representing the ratio of nonfunctional *URA3* clones divided by the total clones obtained from nonselected plates as described in Fig. S2A. Mutations without inactivating the *URA3* function were used to calculate mutational efficiency. The reported values reflect the frequency of *URA3* inactivation rather than the entire mutational efficiency. Furthermore, the control (wild type) and DSB repair mutants were evaluated objectively using the same approach, which can efficiently mitigate the effect of statistical differences in the mutational efficiency to some extent.

When characterizing the possible relationships between DSB repair proteins and genome editing tools, we found that the cell cytotoxicity appeared to correlate with the mutational efficiency. Furthermore, some cases displaying lower cell viability but without an increase in mutation were also observed, such as Dnl4p inactivation under CRISPR/SpCas9-treated conditions (Fig. S6). In this study, we employed a strongly galactose-inducible *GAL1* promoter and a high-copy 2-μ plasmid to control the expression of nucleases, which produced adequate but severe DSB damage. The inefficient NHEJ plays a crucial role in processing DSBs and restoring the DNA integrity without exogenous DNA templates in S. cerevisiae. Once cells are unable to cope with the DSB damage effectively, they can undergo rapid apoptosis. Among a small fraction of surviving cells, the repair outcomes mainly include accurate repair events and mutagenesis. The precisely repaired populations may be subjected to another round of DNA damage under abundant nucleases, while the cells with mutations at the edited locus can effectively survive. Thus, only the mutant cells with a mutation at the edited site could finally survive. To some extent, this observation could possibly explain why cell viability was substantially decreased even in the absence of enhanced mutagenesis.

In this study, AsCpf1 mainly generated 5′ 2-4-nt overhangs and hardly induced insertions in the wild type (Fig. 1D), which was consistent with a previous report (40). However, an AsCpf1 ortholog from *L. bacterium* (LbCpf1) produced mostly 5′ 3-nt overhangs, but no 2-nt overhangs were generated at the cleavage site, and it also caused a small number of insertion events (40). Moreover, S. pyogenes SpCas9 enzymes (SpCas9) created blunt ends as well as a small part of 5′ 1-nt overhangs and induced 20% insertions (40). Thus, we assumed that the generation of microhomology arms facilitates the repair of AsCpf1-induced DSB lesions. The mechanisms underlying events still remain to be explored. We also found that deleting *SAE2* or overexpressing *MRE11*-H125N for DSB end resection showed an improved characteristic of the mutational efficiency, thereby suggesting the “first step resection” could potentially result from these exonucleases and can lead to the different complicated connections with insertional mutations (48). Further characterizing their mutational landscape, the deletion or large deletion events were significantly found to be increased upon modulating *SAE2 and MRE11*. Moreover unexpectedly, the inhibition of exonuclease activity of both Sae2p and Mre11p did not block degradation of DSB ends or cause lesser nucleotide deletion (43). One possible reason for this observation could be that the lesion of exonuclease might have adjusted the DSB checkpoint and recruited other nucleases to effectively process the break ends (31). Moreover, overexpressing *MRE11*-P110L also displayed the higher ratio of nucleotide deletion or large deletion, thereby implying that the increased turnover of the mutated Mre11p might prefer to bind and process DSB in combination with other nucleases (31). Moreover, deleting exonuclease Exo1p for extensive resection also markedly enhanced mutagenesis and increased the ratio of insertion, thereby suggesting that Exo1p indeed can perform the function of 5′–3′ nucleolytic degradation to identify a matching sequence (49). In addition, Fun30p, described as an activator of resection (50), showed similar repair outcomes to Exo1p, which further indicated that the second step of resection might be beneficial to introduce additional mutations. When TALENs were employed to introduce the mutations at *URA3* loci, we unexpectedly found that both the deletion and overexpression of *EXO1* and *FUN30* significantly decreased the mutational efficiency. It appeared that the stoichiometry of Exo1p and Fun30p might be critical for regulating the function of TALENs but not for SpCas9 and AsCpf1. However, the potential effect of Exo1p and Fun30p on TALENs, SpCas9, and AsCpf1 needs to be further explored. In addition, it was noted that the different mutational efficiencies by modulating DSB repair proteins were enhanced in yeast, but still not high enough (less than 13%, one round of genome editing). We speculated that the predetermined checkpoint was developed to respond to the various DNA lesions during the course of evolution (35, 36), and this would substantially hamper the improvement of the mutational efficiency through engineering a couple of DNA repair-related proteins (35). Furthermore, overexpression of Cdc9p, a crucial ligase for imprecise end joining, markedly improved CRISPR/SpCas9-induced mutation efficiency by 6.5 times. *DNL4* deletion, on the other hand, reduced the mutational efficiency by 1.9-fold. As previously reported, Dnl4 can effectively promote mutagenic end-joining independently of its catalytic activity, most likely through a mechanism involving Cdc9p (51). Thus, co-overexpression of *DNL4* and *CDC9* may enhance SpCas9-induced mutagenesis, and this work is worth exploring in the future. Moreover, *rad51* null mutant strain appeared to improve the capacity of CRISPR/AsCpf1 and CRISPR/SpCas9-N863A, but not TALENs. Inactivation of Rad51p in DNA recombination can result in the accumulation of DSBs and reduce the efficiency of HDR (52). However, the overhang pattern may not be an important influencing factor for the *rad51* null mutant, because CRISPR/AsCpf1 and TALENs can also induce similar DSB ends. Thus, the diverse factors that can possibly influence differential effects of *rad51* null mutation on CRISPR/AsCpf1- and TALENs-mediated mutagenesis remain to be uncovered. To varying degrees, CRISPR/AsCpf1, TALENs, and CRISPR/SpCas9 N863A all appear to benefit from the inactivation of Msh2p. Furthermore, both inactivation of DSB ends resection proteins and Msh2p can increase CRISPR/AsCpf1-induced multiple-mismatch. These findings suggest that eliminating nuclease activities of the various resection proteins may induce the mismatch repair (MMR) pathway to repair CRISPR/AsCpf1-induced DSB lesions. Here, we have mainly focused on a part of the repair proteins, which play significant roles in regulating the primary repair pathways. However, more extensive repair factors that have been reported to be involved in damage sensing, signal transduction, cell cycle regulation, and DNA repair to maintain genome stability were not investigated. For example, the helicase Sgs1p and the nuclease/helicase Dna2p can also process the DSB end resection with the existence of replication protein A (RPA) (53). Rad9p, also known as DNA damage checkpoint mediator, plays an important role in causing DSB nucleolytic resection (54). Hence, much more effort in modulating DNA repair could be made to further enhance the capability and mutagenic outcomes of the genome editing in the future.

We also investigated the potential relationship between the cell viability and mutational efficiency, and discovered that the mutational efficiency was inversely proportional to cell viability in most of the mutant strains. Furthermore, the association between the cell survival rate and mutation efficiency varied to a great extent depending on the genome editing technology used (Fig. S6). Thus, further research is necessary to understand the complex mechanism behind the combined effects of the genome editing tools and the various repair factor modifications involved in the regulation of mutation efficiency and cell survival changes. In addition, to evaluate the mutagenesis capacity of the inefficient TALENs, both single sugar (2% galactose) and mixed sugars (2% glucose with 2% galactose) strategies were investigated. Rather than distinguishing between the four tools, the primary goal of this study was to characterize the interaction relationship between the genome editing tools and various DNA repair proteins that can significantly improve the mutational efficiency and landscape for diversifying gene expression. As a result, the differences in the culture methods among the different genome editing tools should be minimized.

Many novel approaches for genome editing have been developed in recent years, thus providing powerful platforms to significantly improve the various cellular phenotypes (4, 55, 56). In this study, the strategy of modulating DSB repair remarkably reshaped the capability of template-free genome editing and especially improved the mutational diversity by about 3-fold. This new strategy could successfully be used to produce the desired phenotypes, such as diversifying the fluorescence expression and decreasing glycerol formation in yeast. Notably, the different DNA repair proteins employed to enhance the template-free editing in this study mainly include Mre11p, Sae2p, Exo1p, and Fun30p, which served to both respond to and repair DSB lesions. In this study, we performed *MRE11*-H125N modification-based CRISPR/AsCpf1 mGE toolkit as a potential proof of concept. The combination of genome editing tools and the modulation of various DNA repair proteins could provide more novel mGE strategies for enhancing genome alterations. For example, selecting *EXO1* or *SAE2* deletion to develop another mGE with CRISPR/SpCas9 or CRISPR/AsCpf1 can be explored in the future as a possible choice.

To validate the enormous promise of the mGE strategy for promoting diversification of gene expression, *eGFP* with the strong and constitutive *Pmini* promoter was used. Although the fluorescence of the mGE-treated population was observed to be diversified, the expression intensity appeared to be significantly weak. The *Pmini* promoter is a strong promoter, and the target site of mGE is positioned at the core elements (upstream activation site; UAS) that can impart robust and constitutive function, which could explain why higher promoter strength was not obtained. In addition, we used mGE to reduce glycerol productivity and then augment ethanol production by targeting the promoter of *GPD1* or *FPS1*. *GPD1* encodes NAD-dependent glycerol-3-phosphate dehydrogenase (GPDH). *FPS1* encodes the aquaglyceroporin, a plasma membrane channel that is involved in the influx and efflux of glycerol. These two genes are important for regulating overall glycerol yield, and their overexpression can increase glycerol yield (57, 58). The mGE edited data confirmed that sgRNAs targeting *FPS1* and *GPD1* created a large dynamic range in glycerol productivity (for *FPS1*, –39.3% to 27.7%; for *GPD1*, –18.3% to 13.5%, respectively) (Fig. S12). Since we sought to reduce the amount of glycerol produced, which is a by-product of ethanol production, only weaker promoter strength mutants were used here. Overall, the results above indicated that mGE can rapidly diversify gene expression, with both lesser and stronger promoter strength as expected. Moreover, while compared to CT-Edit populations, MT-Edit appeared to markedly improve the coefficient of variation at the individual gRNA level only slightly (Fig. 7B). In addition, extra mutation (*MRE11*-H125N) may cause other major problems genome-wide and cause repair issues for off-target sites of the genome editing tool. As a result, whether mGE tools are worthwhile to develop may be debatable. Hence, more diversified mutational landscapes, at least 2-fold against the control, were obtained with *MRE11*-H125N mutation (Fig. 7C). We have reason to also speculate that if the diversity mutation might occur in the promoter's core region, the promoter's expression abundance will be higher, similar to how mGE can enrich eGFP expression (Fig. 6B). Furthermore, *MRE11*-H125N and AsCpf1 were combined to create mGE as a conceptual application. The deletion of *SAE2* or *EXO1* may also be a good option, as these gene deletion mutants have been previously shown to have no significant effect on either the genome architecture or stability (59).

The long-term genome stability and additional detriments of DSB repair protein modifications in the cell were not examined here. Puddu et al. (59) systematically investigated the genome architecture and stability of 4,732 strains comprising the homozygous diploid yeast S. cerevisiae gene-knockout collection (YKOC), and the results clearly indicated that *SAE2*, *EXO1*, or *FUN30* deletion had no significant effects both either the structure or stability of the genome, while the *mre11* deletion and *MRE11*-H125N variant were not studied. Moreover, a loss of Mre11p in S. cerevisiae can result in substantial genomic instability, and sensitivity to the various agents (e.g., DNA damaging agents, short telomeres, defective nonhomologous end-joining [NHEJ], and so on) (60), whereas the *MRE11*-H125N mutation (elimination of the Mre11p nuclease activity) was found to be more resistant to the various DNA damaging agents than *mre11*-deficient cells (61, 62). To eliminate the potential risks from DSB repair protein modifications, complementary approaches could be worth developing. Since CRISPR-based gene manipulation is convenient and efficient in S. cerevisiae (63), it may be a practical choice to complement the defective DSB repair gene in the desired cells that could be generated by the mGE strategy. Moreover, first performing CRISPRi or using small molecules to regulate DSB end resection proteins as well as DNA repair pathways instantaneously, and then employing mGE to diversify genomic alterations, may be another suitable option (56, 64, 65). In summary, the potential risks introduced by DSB repair protein modifications are worthy of further investigation, and could be effectively eliminated by using different complementary technologies.

In this study, three diverse iterative cycles were employed to significantly improve the mutational efficiency up to about 50% (Fig. S9). However, this strategy was mainly suitable for CRISPR/SpCas9 and CRISPR/AsCpf1, but did not work very well for TALENs or CRISPR/SpCas9 N863A due to their low mutational efficiencies. Moreover, mutational efficiency could decrease gradually if more than three cycles were applied (Fig. S9). This observation implied the complicated interplay between genome editing tools and DSB repair machinery, and thus detailed underlying mechanisms remain to be further explored. In addition, genome editing tools have been reported to have multiple off-target effects (38, 66), and it is possible that mGE might also face with similar problems. The use of inducible *GAL* promoter to overexpress nucleases might lead to substantially high off-target rates and cytotoxicity, thus resulting in relatively lower mutational efficiency and cell viability (Fig. 2 and Fig. S8). Nevertheless, the mGE strategy primarily aims to introduce diverse indels, and thus off-target could also provide an unexpected evolutionary driving force to some extent.

Genome editing tools have shown a great potential to drive various gene mutations, corrections, and disruptions for genome engineering (67). In this study, we have systematically characterized the introduction of diversified mutations by using different editing tools based on modulating DSB repair and thereby developed a new strategy of mGE to accelerate genome evolution for obtaining novel or desired phenotypes. Our findings have clearly indicated that not only the types of editing tools but also DSB repair proteins are critical determinants of the repair outcomes in the genome. Since the proteins related to DSB end resection have been found to be evolutionarily conserved in many organisms, the novel mGE approach could be applied to enhance the abundance of the phenotypic diversity to accelerate genome evolution beyond yeast.

## MATERIALS AND METHODS

### Plasmid construction.

All the constructs used in the study have been listed in Table S1. The primers used in this study have been listed in Data Set S3. The ClonExpress II One Step Cloning Kit (Vazyme Biotech Co., Ltd., Nanjing, China) based on the homologous recombination technology was used to construct different plasmids by following the instruction manual. All the plasmids were sequenced by BGI (Beijing, China) for verification.

To mutate S. cerevisiae *URA3* by genome editing tools, the programmable nucleases and the corresponding guide modules were subcloned into the previously reported plasmid p423-GAL-L12 (27, 38), respectively (Fig. 1A). SpCas9 expression cassette was first amplified from pCAS (Addgene) (68) and cloned into the p423-GAL-L12 to replace the TALEN modules to produce p423-GAL-SpCas9. Thereafter, the guide RNA (gRNA) cassette containing the specific gRNA (*URA3*-1) for *URA3* was constructed and inserted into the restriction site *Xma* I of p423-GAL-SpCas9, thereby resulting in the final plasmid p423-gRNA(*URA3*-1)-SpCas9 for CRISPR/SpCas9 editing at *URA3* locus. A codon-optimized *Acidaminococcus* sp. AsCpf1 was amplified from pCDNA3.1-hAsCpf1 (Addgene) (69) and cloned into p423-GAL-SpCas9 to replace SpCas9 coding region, thus resulting in p423-GAL-AsCpf1. Thereafter, CRISPR RNA (crRNA) cassette containing the crRNA scaffold sequence as well as 23-bp specific sequence for *URA3* was constructed. It was then introduced into p423-GAL-AsCpf1 by replacing the specific gRNA of *URA3*-1 and its structural sequence, resulting in the final plasmid p423-crRNA(*URA3*)-AsCpf1 for CRISPR/AsCpf1 editing at *URA3* locus. A TALEN module that can bind to the 12-bp right target sequence was amplified from p425-GAL-R12 (27) and subcloned into the restriction site *Xma* I of p423-GAL-L12 to produce the final plasmid p423-GAL-L12R12 for TALENs editing at *URA3* locus. The point mutation of N863A was introduced into the SpCas9 protein of p423-gRNA(*URA3*-1)-SpCas9 through overlap extension PCR by using the primer pairs of N863A-F/N863A-R. Thereafter, *URA3* specific gRNA of *URA3*-1 was replaced by a new *URA3* specific gRNA of *URA3*-2, and the other gRNA expression cassette containing the specific gRNA (*URA3*-3) for *URA3* was obtained from pCAS by using the standard fusion PCR. It was then inserted into the restriction site *Pst* I, resulting in the plasmid of pRS423-gRNA2-gRNA3-SpCas9 N863A for CRISPR/SpCas9 N863A editing at *URA3* locus. These two gRNA expression cassettes of *URA3*-2 and *URA3*-3 targeted opposite DNA strands respectively at a distance of 48 bp with PAMs in the *URA3* gene. To evaluate the mutational efficiency and mutational types introduced by CRISPR/SpCas9 and CRISPR/AsCpf1 at another target site (*ADE2*), the plasmids p423-gRNA(*ADE2*)-SpCas9 and p423-crRNA(*ADE2*)-AsCpf1 were constructed by replacing the specific guide RNA sequences of p423-gRNA(*URA3*-1)-SpCas9 and p423-crRNA(*URA3*)-AsCpf1.

To construct the plasmids to facilitate the expression of the wild-type or point mutation DSB repair genes, a backbone plasmid containing a *TEF1* promoter and a synthetic terminator *Tsynth8* (70) at the restriction site BamH I was first generated using pRS315, resulting in p315-TEF1-T8. Then, the DNA fragments of *MRE11*-H125N, *MRE11*-P110L, or *SAE2-*S267E, obtained by using standard fusion PCR, and other wild-type DSB repair genes of *FUN30*, *EXO1*, and *CDC9* were inserted into p315-TEF1-T8 under the control of the *TEF1* promoter and *Tsynth8* terminator, respectively. To construct the plasmids to edit the synthetic minimal promoter *Pmini* (Fig. S10) and to regulate *eGFP* expression or the native *FPS1* and *GPD1* promoter by CRISPR/AsCpf1, 23-bp specific crRNA sequence for *URA3* in p423-crRNA(*URA3*)-AsCpf1 was replaced by the *Pmini*-specific crRNA, *FPS1*, or GPD1-specific crRNA in the primer pairs (Data set S3) to obtain the corresponding editing plasmids (Table S1), respectively.

To verify whether the diverse fluorescence phenotypes were due to the mutated synthetic promoters, a series of plasmids containing the different *eGFP* cassettes was constructed. The DNA fragments of *eGFP* cassettes containing mutated or original *Pmini* promoter, *eGFP* coding region, and the *CYC1* terminator were thereafter amplified from the genomic DNA of the mutant strains and BY4741a (*pdc1*Δ*::eGFP*), and were further inserted into the restriction site *Sac* I of pRS315, respectively.

### Strain construction and incubation.

All the strains used in this study (Data Set S4) were derived from S. cerevisiae BY4741a with the functional *URA3* under its own promoter and terminator at the *PDC1* locus (27). The single-gene deletion strains derived from BY4741a were next constructed by using a one-step gene replacement method based on *KanMX* modules flanked by 500-bp homologous sequences (71). To obtain the double deletion strain of *SAE2* and *MRE11* genes, *SAE2* was further replaced with the ZeoR expression cassette in the *mre11*Δ strain. The ZeoR expression cassette was first PCR amplified from the plasmid pIS438 (72), and then fused with approximately 500-bp homologous sequences located at the upstream and downstream of the *SAE2* gene, which was PCR amplified from BY4741a genomic DNA. Two different strains expressing *eGFP* under the control of the synthetic minimal promoter *Pmini* (Fig. S10) and the *CYC1* terminator were next constructed by replacing the *URA3* expression cassette at the *PDC1* locus in S. cerevisiae BY4741a and *mre11*Δ strain, thereby resulting in BY4741a (*pdc1::eGFP*) and BY4741a (*mre11*Δ, *pdc1::eGFP*), respectively. The *Pmini* promoter was synthesized by BGI (Beijing, China). The *eGFP* expression cassette fused with approximately 500-bp homologous sequences located at the upstream and downstream of the *PDC1* gene was obtained by using two different rounds of fusion PCR. All the strains were confirmed by PCR amplification and subsequent DNA sequencing. The parent or derivative strains were transformed with 2 μ plasmid carrying either the genome editing modules (Fig. 1A) and/or centromeric plasmid carrying the target gene for overexpression experiments to potentially test the effects of related genes or mutants on mutational efficiency and diversity. Yeast transformation was performed by using either the standard lithium acetate method (73) or electroporation (74). Positive transformants were then selected and grown on the synthetic defined (SD) media with 20 g/L glucose and the appropriate concentrations of auxotrophic compounds. Parental S. cerevisiae BY4741a and its derivatives were grown in either YPD media (per L, 10 g yeast extract, 20 g peptone, 20 g glucose) or SD media with the appropriate auxotrophic compounds and/or 5-fluoroorotic acid (5-FOA, a dose of 0.15%) in the specified experiments (74).

### Characterization of *URA3* and *ADE2* mutational efficiency and diversity.

S. cerevisiae BY4741a and the various mutants (described in Data Set S4) were applied for the *URA3* mutagenesis experiment with the four different genome editing tools to evaluate the modification efficiency and diversity at the *URA3* locus. The control plasmids of pRS423 and pRS315 were also used to transform S. cerevisiae BY4741a as a negative control without carrying out the genome editing. The transformants were cultured in SD media containing 20 g/L glucose and the appropriate concentrations of auxotrophic compounds to an early logarithmic stage. They were then inoculated at optical density of the cultures at 600 nm (OD_600_) of 0.4 into SD media containing 20 g/L galactose and the appropriate auxotrophic compounds for a further 36 h. Next, the cell cultures were plated on SD media with the appropriate auxotrophic compounds in the presence or absence of 5-FOA. The cells with the mutated and nonfunctional *URA3* can grow on 5-FOA plates, whereas the cells with functional *URA3* are unable to grow (75). In the case of *URA3*, the mutational efficiency (exactly, *URA3* inactivation frequency) introduced by the various genome editing tools in the control (wild type) and DSB repair mutants was calculated from colony numbers obtained on SD + 5-FOA agar plates versus SD agar plates, as described in the main text and Fig. S2A. Thus, the mutational efficiency was expressed as a percentage is the proportion of nonfunctional *URA3* clones to total clones (nonmodified clones plus mutated clones yet with intact *URA3* function) (Fig. S2A). To identify the mutational diversity induced by the potential interplay between the genome editing tools and DSB repair proteins, the *URA3* fragment was amplified from these 5-FOA-resistant colonies and the primer pair *URA3*-F/*URA3*-R (Data Set S3), and sequenced using the primer *URA3*-Seq for further alignment analysis. To test whether mutational efficiency could be improved through serial transfer in the liquid media, serial transfers every 24 h for the total five rounds were conducted for the wild-type strain with p423-gRNA(*URA3*-1)-SpCas9, p423-crRNA(*URA3*)-AsCpf1, or p423-GAL-L12R12 to edit the *URA3* gene. The mutational efficiency was thereafter measured and calculated as described above. In the case of *ADE2*, the mutational efficiency (exactly, *ADE2* inactivation frequency) introduced by CRISPR/SpCas9 and CRISPR/AsCpf1 was calculated from red colony numbers obtained on SD + 5 mg/L adenine agar plates (2% galactose induction) versus total colony numbers obtained on SD + 5 mg/L adenine agar plates (2% galactose induction) (Fig. S2B). We also performed amplicon sequencing to further characterize the mutational diversity.

### Amplicon sequencing.

To completely characterize the different genetic variations generated by CRISPR/SpCas9 and CRISPR/AsCpf1 in wild-type and various mutants (Data Set S4) at *URA3* and *ADE2* loci, amplicon sequencing technology was carried out. The library preparation and amplicon sequencing were conducted by Novogene Co., Ltd. (Beijing, China). To identify the various mutations in amplicon sequencing data, MUSCLE (Version 3.8.31, http://www.drive5.com/muscle/) was performed for the multiple alignments, and then DNA MAN (version 5.2) software was used to manually proofread the data. Similarly, the amplicon sequencing technology was used to investigate the mutational types at *FPS1* and *GPD1* gene promoters introduced by the mGE toolkit.

### Diversified fluorescence expression analysis.

S. cerevisiae BY4741a (*pdc1*::*eGFP*) (CT) and BY4741a (*mre11*Δ, *pdc1*::*eGFP*)/p*MRE11*-H125N (MT) carrying CRISPR/AsCpf1 expression plasmids were grown overnight in SD media with 20 g/L glucose and the appropriate concentration of auxotrophic compounds. One aliquot of the overnight cultures was then transferred into fresh SD media with 20 g/L galactose along with the appropriate auxotrophic compounds, and then incubated for 24 h. After the three cycles of serial transfer, the cell cultures were analyzed and sorted by a BD Influx flow cytometer (Franklin Lakes, NJ, USA). An average fluorescence and standard deviation were calculated to characterize fluorescence diversity. The mutants with diverse phenotypes were next selected to further measure in triplicates as described above and thereafter sequenced to confirm the genetic variation at the target locus after PCR amplification. To reconfirm the diverse fluorescence phenotypes, the strains expressing *eGFP* under the control of the mutated or original synthetic minimal promoters were first constructed by transforming *eGFP* expressing plasmids of p315-Pmini(R1)-GFP, p315-Pmini(R2)-GFP, p315-Pmini(R3)-GFP, p315-Pmini(R4)-GFP, p315-Pmini(R5)-GFP, p315-Pmini(R6)-GFP, and p315-Pmini(Original)-GFP into S. cerevisiae BY4741a to assess the possible expression levels of the *eGFP*. The fluorescence value was measured using a Spectramax M2 microplate reader (Molecular Devices, USA) with excitation at 488 nm and emission wavelengths at 509 nm, and normalized to the cell density by measuring the optical density of the cultures at 600 nm (OD_600_).

### Glycerol productivity analysis based on mGE.

The plasmids with the specific guide and nuclease module targeting native promoter of *FPS1* and *GPD1* were first transformed into S. cerevisiae *mre11*Δ mutant strain harboring p*MRE11*-H125N (MT) for diversifying glycerol productivity. S. cerevisiae BY4741 harboring pRS423 (CT) was used as the control. The blank plasmids pRS315 and pRS423 without nucleases modules were transformed into BY4741 and derived *MRE11*-H125N mutant strain to generate the control strains for evaluating the background of the population diversity. The transformants were then cultured as described above. After three cycles of the serial transfer, the evolutionary cell populations were appropriately diluted and plated on the SD media with 20 g/L glucose and the appropriate auxotrophic compounds to ensure the single-colony isolation. After 48 h of incubation, about 100 single colonies were randomly picked and cultivated in the SD media with 20 g/L glucose and the appropriate auxotrophic compounds for 24 h in 96 deep well plates. The induction and screening process was repeated for another three cycles, and the colonies were picked and compared by measuring the glycerol productivity with the optimized colorimetric method (76). The parallel strain without CRISPR/AsCpf1 editing was used as a negative control. Glycerol productivity diversity was characterized as the coefficient variation (CV).

### Fermentation assays.

To evaluate the fermentation capacity of the two generated mutants (FPS1-M and GPD1-M), ethanol fermentation experiments were conducted in 100-mL Erlenmeyer flasks containing 50 mL SD medium with 100 g/L glucose and appropriate concentrations of auxotrophic compounds at 220 rpm. The cells were precultured in SD medium at 30°C for overnight before being used for the fermentation experiments. The starting OD_600_ used in all the experiments was 0.2. Optical density (OD) at 600 nm was measured using a plate reader (Molecular Devices SpectraMax M2e, San Jose, CA, USA). The concentrations of the glucose and ethanol were monitored by high-performance liquid chromatography (HPLC) using an Agilent 1260 system (Agilent, Santa Clara, CA, USA) equipped with a refractive index detector and a Phenomenex RFQ fast acid column (100 mm × 7.8 mm ID) (Phenomenex Inc., Torrance, CA, USA). The column was then eluted with 0.01 N H_2_SO_4_ at a flow rate of 0.6 m/min at 55°C.

### Data availability.

The data generated in this study have been submitted to the NCBI BioProject database (https://www.ncbi.nlm.nih.gov/bioproject/) under accession number PRJNA753971. Other supplementary data are available at Microbiology Spectrum online.
