# Supplementary material for: Modulating DNA Repair Pathways to Diversify Genomic Alterations in Saccharomyces cerevisiae
Source: Microbiol Spectr. 2022 Mar 30;10(2):e02326-21. doi: 10.1128/spectrum.02326-21 (PMC9045378; doi:10.1128/spectrum.02326-21)
Supplement: SUPPLEMENTAL FILE 5 — Supplemental material. Download SPECTRUM02326-21_Supp_5_seq14.pdf, PDF file, 2.1 MB [file spectrum02326-21_supp_5_seq14.pdf]

Supplementary Material for:

Modulating DNA repair pathways to diversify genomic alterations in *Saccharomyces cerevisiae*

Zhen Wang[1,2], Yuping Lin[1,3], Zongjie Dai[1,3], Qinhong Wang[1,3#]

[1]CAS Key Laboratory of Systems Microbial Biotechnology, Tianjin Institute of Industrial Biotechnology, Chinese Academy of Sciences, Tianjin 300308, China

[2]College of Science & Technology, Hebei Agricultural University, Cangzhou, Hebei, 061100, China

[3]National Center of Technology Innovation for Synthetic Biology, Tianjin, 300308, China.

Running Head: Reshaping DSB repair to enhance genome editing

# Address correspondence to Qinhong Wang, wang_qh@tib.cas.cn

Zhen Wang and Yuping Lin contributed equally to this article. Author order was determined by the corresponding author after negotiation.

The Supplementary materials include:

Table S1. Plasmids used in this study.

Figure S1. The diagram of DSBs generated by different genome editing tools in this study.

Figure S2. The different approaches used for evaluating cell viability, mutation efficiency and diversity after genome editing.

Figure S3. The mutational landscapes generated by the different genome editing tools with wild type DSB repair machinery at *URA3* locus.

Figure S4. Schematic of DSB repair system in *S. cerevisiae*.

Figure S5. Effect of modulating DSB repair proteins on the mutation efficiency (A) and cell viability (B) of four genome editing tools at *URA3* locus.

Figure S6. Cell viability versus mutational efficiency for four genome editing tools in wild type and various DSB repair-related gene mutant strains.

Figure S7. Effect of modulating end resection proteins on the mutation efficiency and diversity at *ADE2* locus.

Figure S8. Effect of modulating DSB repair factors proteins on the mutational efficiency and cell viability with TALENs.

Figure S9. Improving the mutational efficiency of CRISPR/SpCas9, CRISPR/AsCpf1 and TALENs via iterative editing.

Figure S10. The DNA sequence of the synthetic minimal promoter *Pmini*.

Figure S11. Mutational diversity of the fluorescence expression and genome mutagenesis resulted from the synthetic promoter editing via mGE in wild type.

Figure S12. Characterization of the diversity of glycerol productivity resulted from pFPS1 and pGPD1 editing via mGE with three guides.

Figure S13. Evaluation of the fermentation properties of FPS1-M and GPD1-M.

Dataset S1. The mutation types at the *URA3* loci in wild type and various DSB repair mutant strains generated by four genome editing tools form 100 random sequencing events.

Dataset S2. The mutation types at *URA3*, *ADE2*, pFPS1 and pGPD1 detected by amplicon sequencing

Dataset S3. The primers used in this study.

Dataset S4. The strains used in this study.

Table S1. Plasmids used in this study

Plasmid	Description	Reference or source
pRS423	Amp+, *HIS3*, 2μ origin	Invitrogen
pRS315	Amp+, *LEU2*, ARS/CEN origin	Invitrogen
p423-GAL-L12	pRS423 with TALEN module binding to L12 of *URA3*, pGAL1	(1)
p425-GAL-R12	pRS425 with TALEN module binding to R12 of *URA3*, pGAL1	(1)
)pCAS	Expresses *S. pyogenes* SpCas9 plus an HDV ribozyme-sgRNA for genome editing in yeast	(2)
pCDNA3.1-hAsCpf1	Expresses humanized AsCpf1	(3)
pIS438	Containing a yeast replication origin, the counter/selectable *URA3* gene and the dominant marker *ZEO*	(4)
p423-GAL-SpCas9	pRS423 expressing SpCas9-NLS under the *GAL1* promoter and *CYC1* terminator	This study
p423-gRNA(*URA3*-1)-SpCas9	pRS423 containing SpCas9 and the *URA3* specific gRNA (*URA3*-1) expression cassettes	This study
p423-GAL-AsCpf1	pRS423 expressing AsCpf1-NLS under the *GAL1* promoter and *CYC1* terminator	This study
p423-crRNA(*URA3*)-AsCpf1	pRS423 containing AsCpf1 and the *URA3* specific crRNA expression cassettes	This study
p423-GAL-L12R12	pRS423 containing TALEN modules that recognize the 12-bp left and right target sequences with a 22-bp spacer length in the *URA3* gene	This study
p423-gRNA(*URA3*-1)-SpCas9 N863A	pRS423 containing the SpCas9 N863A and the *URA3* specific gRNA (*URA3*-1) expression cassettes	This study
p423-gRNA1-gRNA2-SpCas9 N863A	pRS423 containing two URA3 specific gRNA expression cassettes and expressing SpCas9 nickase variant (SpCas9 N863A) under the *GAL1* promoter and *CYC1* terminator	This study
p423-gRNA(*ADE2*)-SpCas9	pRS423 containing SpCas9 and the *ADE2* specific gRNA (*ADE2*) expression cassettes	This study
p423-crRNA(*ADE2*)-AsCpf1	pRS423 containing AsCpf1 and the *ADE2* specific crRNA expression cassettes	This study

p315-TEF1-T8	Amp$^+$, *LEU2*, ARS/CEN origin, containing the *TEF1* promoter and the Tsynth8 terminator, used for making the constructs to express wild type or point mutant DNA repair proteins	
p*MRE11*-P110L	pRS315 expressing *MRE11-P110L*	This study
p*MRE11*-H125N	pRS315 expressing *MRE11-H125N*	This study
p*SAE2*-S267E	pRS315 expressing *SAE2-S267E*	This study
p*FUN30*	pRS315 expressing *FUN30*	This study
p*EXO1*	pR315 expressing *EXO1*	This study
p*CDC9*	pRS315 expressing *CDC9*	This study
p423-crRNA(Pmini)-AsCpf1	CRISPR/AsCpf1 genome editing plasmid containing the specific crRNA for *Pmini* to target the synthetic minimal promoter of *eGFP*	This study
p423-crRNA(*FPS1*-1)-AsCpf1	CRISPR/AsCpf1 genome editing plasmid containing the specific crRNA (*FPS1*-1) for the *FPS1* promoter	This study
p423-crRNA(*FPS1*-2)-AsCpf1	CRISPR/AsCpf1 genome editing plasmid containing the specific crRNA (*FPS1*-2) for the *FPS1* promoter	This study
p423-crRNA(*FPS1*-3)-AsCpf1	CRISPR/AsCpf1 genome editing plasmid containing the specific crRNA (*FPS1*-3) for the *FPS1* promoter	This study
p423-crRNA(*GPD1*-1)-AsCpf1	CRISPR/AsCpf1 genome editing plasmid containing the specific crRNA (*GPD1*-1) for the *GPD1* promoter	This study
p423-crRNA(*GPD1*-2)-AsCpf1	CRISPR/AsCpf1 genome editing plasmid containing the specific crRNA (*GPD1*-2) for the *GPD1* promoter	This study
p423-crRNA(*GPD1*-3)-AsCpf1	CRISPR/AsCpf1 genome editing plasmid containing the specific crRNA (*GPD1*-3) for the *GPD1* promoter	This study
p315-Pmini(R1)-GFP	pR315 expressing mutated *GFP* cassette, which was amplified from R1 mutant genome DNA	This study
p315-Pmini(R2)-GFP	pR315 expressing mutated *GFP* cassette, which was amplified from R1 mutant genome DNA	This study
p315-Pmini(R3)-GFP	pR315 expressing mutated *GFP* cassette, which was amplified	This study

	from R3 mutant genome DNA	
p315-Pmini(R4)-GFP	pR315 expressing mutated *GFP* cassette, which was amplified from R4 mutant genome DNA	This study
p315-Pmini(R5)-GFP	pR315 expressing mutated *GFP* cassette, which was amplified from R5 mutant genome DNA	This study
p315-Pmini(R6)-GFP	pR315 expressing mutated *GFP* cassette, which was amplified from R6 mutant genome DNA	This study
p315-Pmini(Original)-GFP	pR315 expressing *GFP* driven by the original synthetic minimal promoter *Pmini*	This study

Genome editing tool	Cleavage patterns	Diagram of double strand breaks after cleavage
CRISPR/SpCas9	Blunt（Dominant）<br><br>5' 1-nt overhang	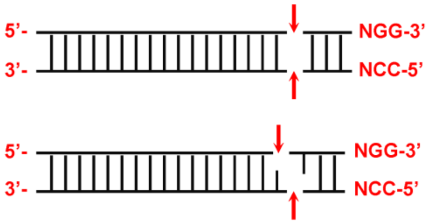
CRISPR/AsCpf1	5' 2～4-nt overhang	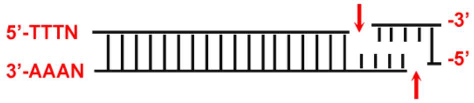
TALENs	5' 4-nt overhang	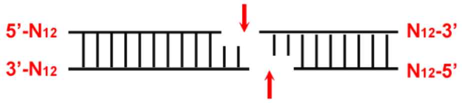
CRISPR/SpCas9 N863A	3' N^1-nt overhang	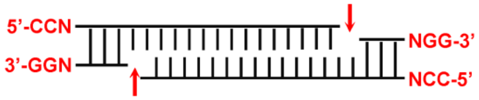

Figure S1. The diagram of DSBs generated by different genome editing tools in this study. CRISPR/SpCas9 typically recognized canonical NGG PAMs and primarily produced blunt end at ~ 3-4 bp upstream of PAM as well as 1-nt 5' overhang at low frequency. CRISPR/AsCpf1 exhibited activity on the site with NTTT and generated 5' 2~4 nts overhang at the tip of guide sequence. TALENs usually employed 12 tandem repeats pairs to bind to DNA and can exploit FokIs to introduce 4-nts 5' overhang. CRISPR/SpCas9 N863A without HNH domain activity produced 3' overhang with two suitably designed guides. N^1: programmable designed number. The number of nucleotides can be artificially designed to meet specific requirements, in this study, 48-bp; N_{12}: twelve random nucleotides.

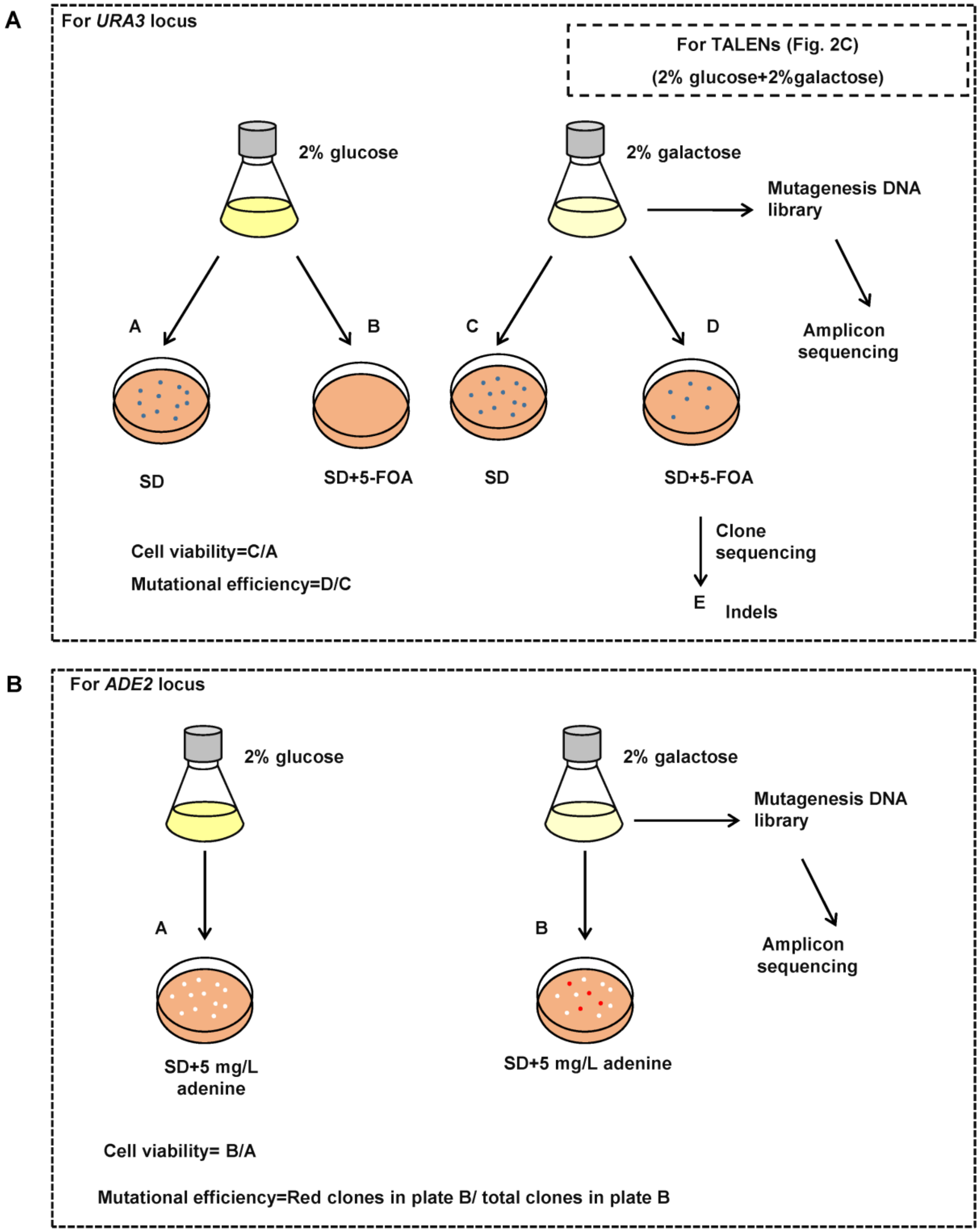

Figure S2. The different approaches used for evaluating cell viability, mutation efficiency and diversity after genome editing. The cell viability introduced by four genome editing tools was calculated based on the colony number in SD agar plated with edited yeast cell culture (2% galactose induction) dividing the colony number in SD agar plated with non-edited yeast cell culture (2% glucose as control). The mutational

efficiency of *URA3* generated by four editing tools was calculated based on the colony number in SD+5-FOA agar plated with edited yeast cell culture (2% galactose induction) dividing the colony number in SD agar plated with edited yeast cell culture (2% galactose induction). For TALENs, a mixed strategy (2% glucose and 2% galactose) was also performed to evaluate the cell viability and mutational efficiency. The mutational landscapes and diversity were analyzed based on the sequencing results of target loci from 100 randomly selected out 5-FOA-resistant mutants (edited mutants) for each genome editing. To investigate the mutational types, genomic DNA of edited populations were extracted and mixed with an equal dose for amplicon sequencing. The mutational efficiency of *ADE2* generated by CRISPR/SpCas9 and CRISPR/AsCpf1 was calculated based on the red colony number in SD+5 mg/L adenine agar plated with edited yeast cell culture (2% galactose induction) dividing the total colony number in SD agar plated with edited yeast cell culture (2% galactose induction). The mutational diversity was analyzed based on amplicon sequencing.

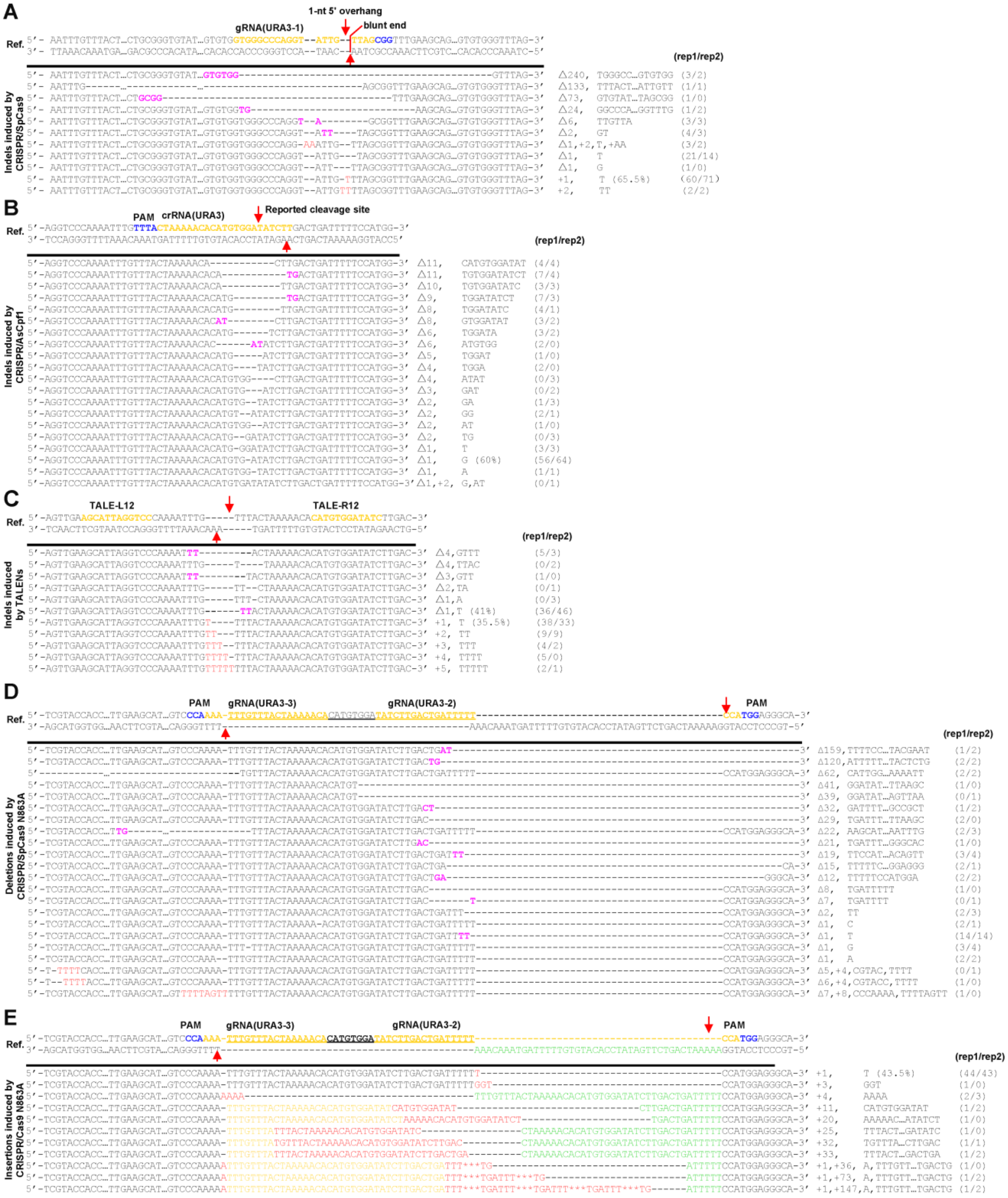

Figure S3. The mutational landscapes generated by the different genome editing tools with wild type DSB repair machinery at *URA3* locus. The mutational landscape was analyzed from each 100 mutants generated by the different genome editing tools as described in Figure S2. PAM sites have been indicated

in bold and blue. Guide sequences have been indicated in orange. Red arrows were used to indicate the potential cleavage sites of nucleases of the different genome editings. Deletion and insertion mutations have been shown using dash line and red sequence, respectively. Microhomology sequences have been indicated in pink. The size and sequence of indels were presented in the right end. The frequency of each mutation in repeat 1 and repeat 2 has been shown on the right. The percentages of the various dominant mutations have been also shown. In (e), the 42-bp 3' overhang generated in Watson strand was shown in the bold and underlined, whereas the reverse complementary 3' overhang generated in Crick strand was shown in green. The *** symbol represents the omitted sequence of insertions: 5'-GTTTACTAAAAACACATGTGGATATCT -3'.

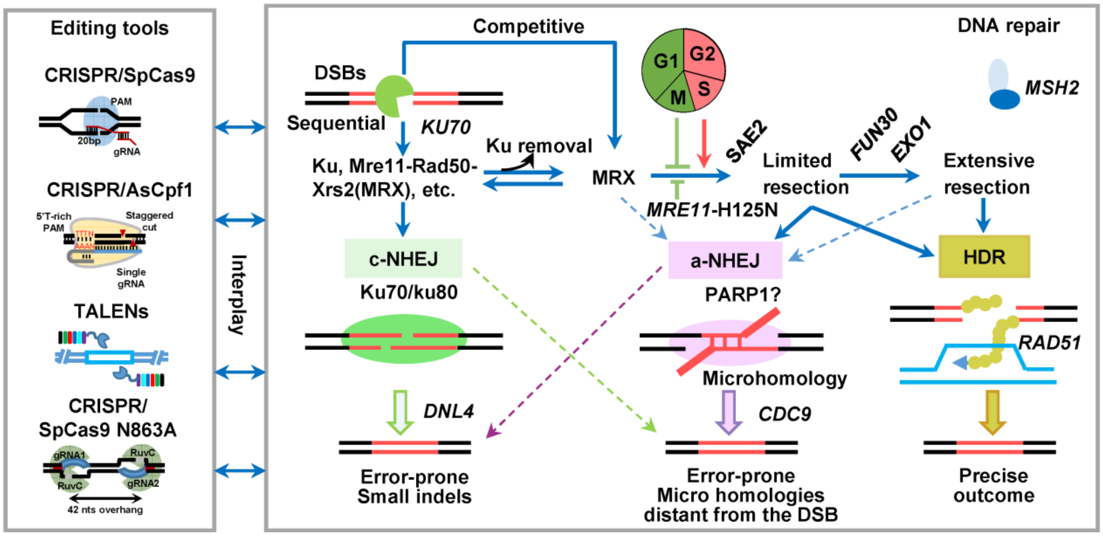

Figure S4. Schematic of DSB repair system in *S. cerevisiae*. The genome editing tools (CRISPR/SpCas9, CRISPR/AsCpf1, TALENs and CRISPR/SpCas9 N863A) induced DSBs at the target locus, and then the different DSB repair mechanisms, canonical and alternative non-homologous end joining NHEJ (c-NHEJ and a-NHEJ) as well as homology directed repair (HDR) were activated in response to this lesion for facilitating the seal breaks to maintain the genome stability. The key genes for DSB repair proteins were noted. Ku70p is the first protein of NHEJ that binds to a broken DNA end and protects it from nucleolytic degradation. *DNL4* encodes DNA ligase IV, which was strictly required in non-homologous end joining. *The Mre11–Rad50–Xrs2* (MRX) nuclease/ATPase complex plays an important structural and catalytic role in the pathways mentioned above. The *MRE11-H125N* mutant strain is deficient for nuclease activity but maintains the ability to form MRX. The *MRE11-P100L* mutant strain increases the turnover of Mre11p from the damaged sites *in vivo*. Sae2p initiates 5'-3' end resection, and also takes an active part in the damping of DNA damage signaling. *CDC9* encodes DNA ligase III, which is essential for a-NHEJ. *EXO1*, encoding 5'-3' exonuclease, processes the limited resection intermediate to generate an extensive resection of ssDNA for HR components. *FUN30*, encoding ATPase, can physically bind to DSB ends and directly promote Exo1p-dependent end resection. Rad51p, also known as RecA, can function to search for the homologous template DNA during the repair of DSBs, which is a critical step in HR. *MSH2* encodes a conserved MutS homologues (MSH) family protein, which involves in DNA mismatch repair.

A Fold change of mutational frequency at *URA3* locus (versus wild type strain)

	WT	*ku70Δ*	*mre11Δ*	*MRE11-P110L*	*MRE11-H125N*	*sae2Δ*	*SAE2-S267E*	*exo1Δ*	*fun30Δ*	*EXO1-OE*	*FUN30-OE*	*dnl4Δ*	*CDC9-OE*	*sae2Δ, MRE11-H125N*	*ku70Δ, CDC9-OE*	*rad51Δ*	*msh2Δ*
CRISPR/SpCas9	1.0	-2.8	-∞	1.5	1.3	2.5	-1.5	13.5	22.7	1.9	-1.7	-3.0	6.5	1.3	-1.2	1.1	-1.2
CRISPR/AsCpf1	1.0	-4.9	-∞	-2.7	6.4	30.2	-17.0	2.4	4.0	1.1	-1.9	-565.2	-2.7	4.6	-58.7	2.2	8.8
TALENs	1.0	-17.1	-15.2	1.0	2.7	1.5	-49.1	-2.5	-1.9	-13.9	-25.9	-9.1	-1.8	2.3	-11.5	-4.5	2.1
CRISPR/SpCas9 N863A	1.0	-1.1	-∞	2.6	-1.4	3.3	-1.0	2.0	1.4	-1.4	1.6	-1.3	1.2	2.8	1.1	3.7	4.0

End protection: *ku70Δ*
End tether and scaffold: *mre11Δ*, *MRE11-P110L*, *MRE11-H125N*
End resection: *mre11Δ* – *FUN30-OE*
End ligation: *dnl4Δ*, *CDC9-OE*
DNA recombination: *rad51Δ*
mismatch correction: *msh2Δ*

Fold change: ≤-2 -1 1 ≥2

B Fold change of cell viability at *URA3* locus (versus wild type strain)

	WT	*ku70Δ*	*mre11Δ*	*MRE11-P110L*	*MRE11-H125N*	*sae2Δ*	*SAE2-S267E*	*exo1Δ*	*fun30Δ*	*EXO1-OE*	*FUN30-OE*	*dnl4Δ*	*CDC9-OE*	*sae2Δ, MRE11-H125N*	*ku70Δ, CDC9-OE*	*rad51Δ*	*msh2Δ*
CRISPR/SpCas9	1.0	-1.8	-8.9	-1.1	1.7	22.8	-14.8	-1.8	-2.8	1.0	3.9	-2.2	3.1	1.3	-1.3	-2.1	1.8
CRISPR/AsCpf1	1.0	1.1	-4.3	1.0	-1.2	1.7	2.1	2.4	-2.9	1.6	2.8	47.5	2.7	-1.2	4.5	-3.6	-1.4
TALENs	1.0	1.4	-1.9	1.2	1.3	-1.1	2.4	1.8	1.2	1.6	1.2	-1.3	1.6	2.1	1.1	2.3	1.1
CRISPR/SpCas9 N863A	1.0	-2.4	-13.1	-2.6	-3.2	-3.4	-1.0	-4.0	-2.5	-2.2	-2.5	-1.6	1.2	-4.4	-2.8	1.2	-2.3

End protection: *ku70Δ*
End tether and scaffold: *mre11Δ*, *MRE11-P110L*, *MRE11-H125N*
End resection: *mre11Δ* – *FUN30-OE*
End ligation: *dnl4Δ*, *CDC9-OE*
DNA recombination: *rad51Δ*
mismatch correction: *msh2Δ*

Figure S5. Effect of modulating DSB repair proteins on the mutation efficiency (A) and cell viability (B) of four genome editing tools at *URA3* locus. CRISPR/SpCas9, CRISPR/AsCpf1, TALENs and CRISPR/SpCas9N863A-induced mutation efficiency and the cell viability were evaluated in various DSB repair related gene mutant strains and the fold change was analyzed. The fold change of mutational efficiency and cell viability in WT strain was normalized as 1.0 corresponding to each genome editing tool, and others were calculated as the values related to those of WT, respectively.

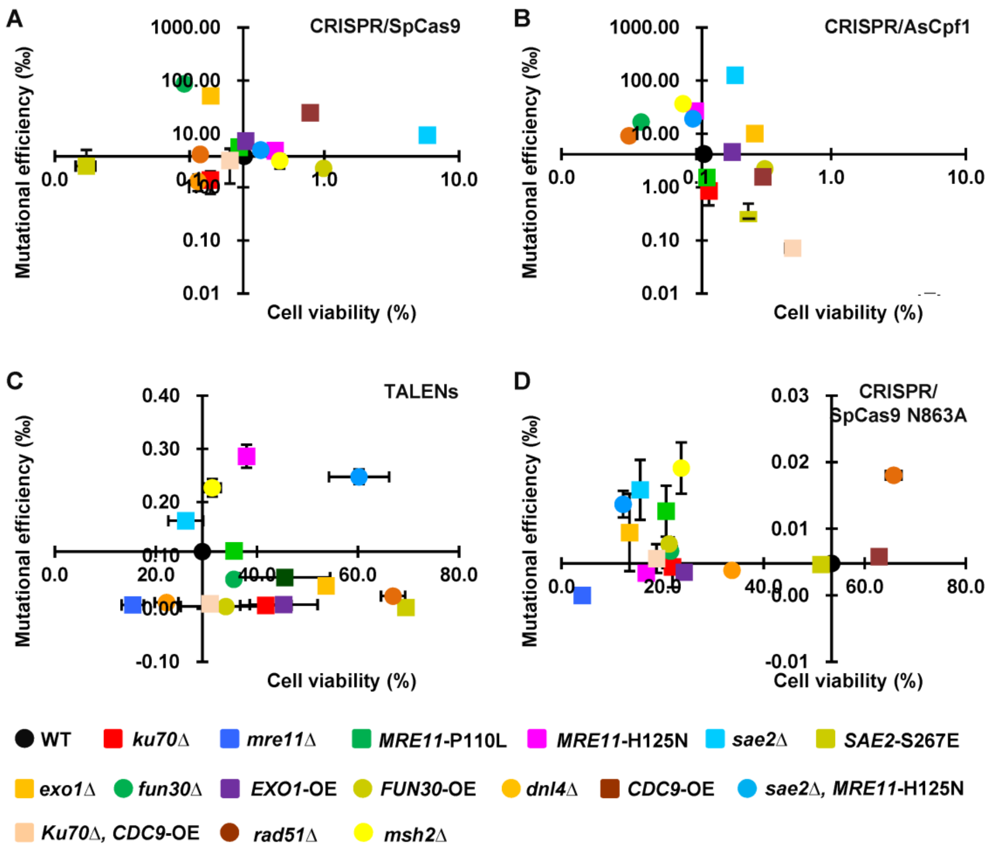

Figure S6. Cell viability versus mutational efficiency for four genome editing tools in wild type and various DSB repair-related gene mutant strains. (A) CRISPR/SpCas9, (B) CRISPR/AsCpf1, (C) TALENs, and (D) CRISPR/SpCas9N863A-induced mutation efficiency and the cell viability were evaluated and compared. Notably, the TALENs were performed with 2% galactose and 2% glucose. The mutational efficiency and cell survival ability was calculated using the method described in Figure S2.

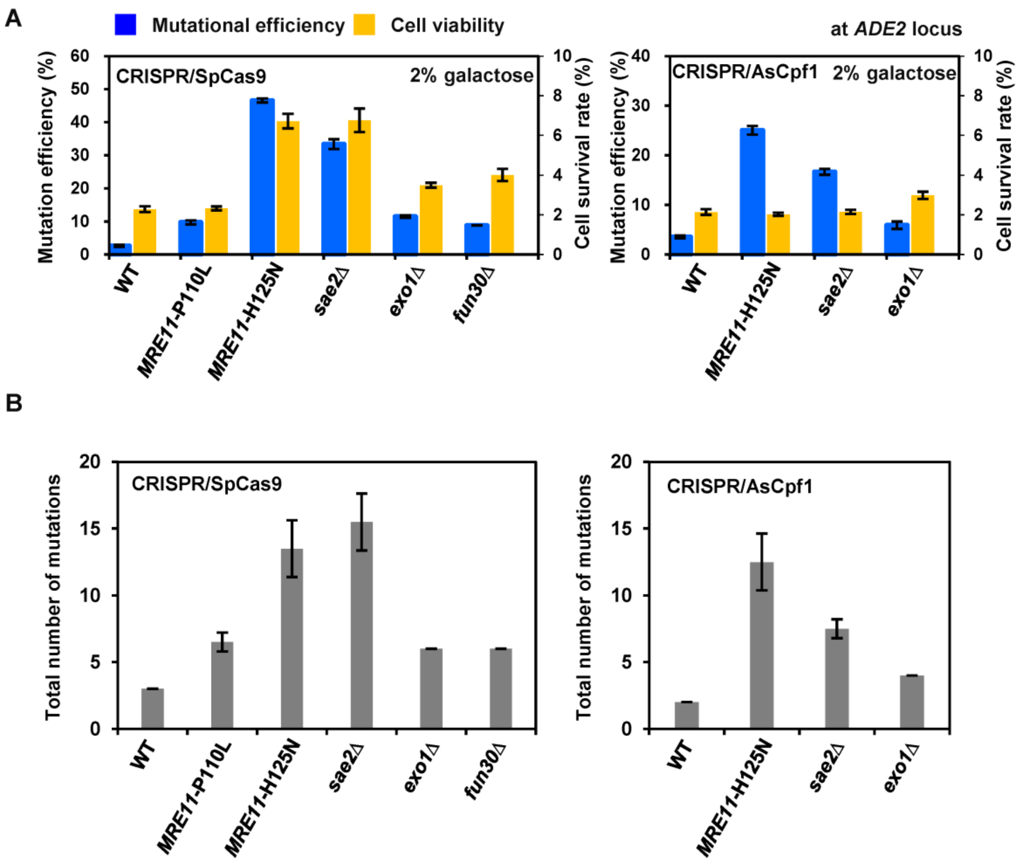

Figure S7. Effect of modulating end resection proteins on the mutation efficiency and diversity at *ADE2* locus. The CRISPR/SpCas9 and CRISPR/AsCpf1 based mutation efficiency and the cell viability (A) was evaluated through the approach described as Figure S2, and the mutation diversity (B) was characterized using amplicon sequencing as described as methods.

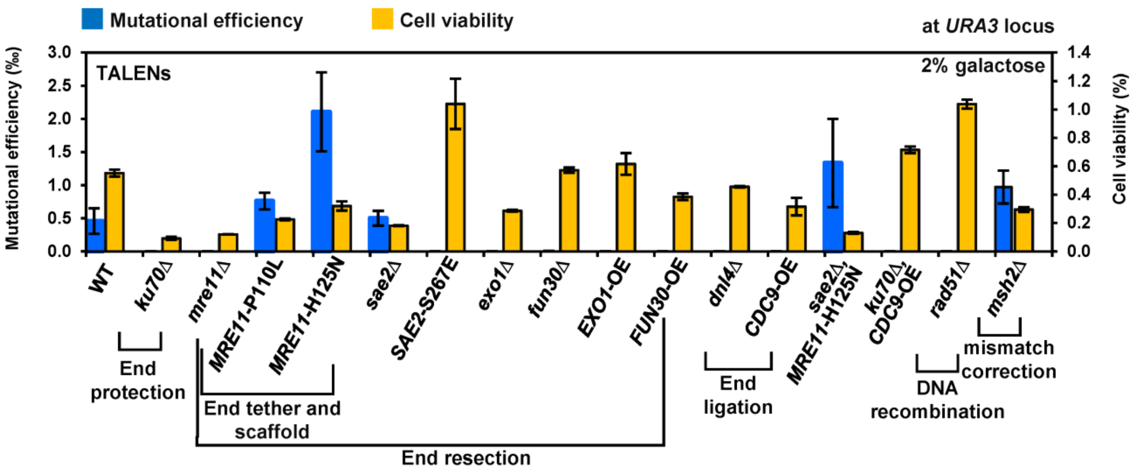

Figure S8. Effect of modulating DSB repair proteins on the mutational efficiency and cell viability with TALENs. TALENs-induced mutational efficiency and the cell viability were evaluated in various DSB repair related gene mutant strains. Notably, the TALENs were performed with 2% galactose. Blue column: mutational efficiency; Yellow column: cell viability. The mutational efficiency and cell survival ability was calculated using the method described in Figure S2. All the strains contained the plasmids with different the programmable nucleases and guide modules for the different genome editing (Figure 1A).

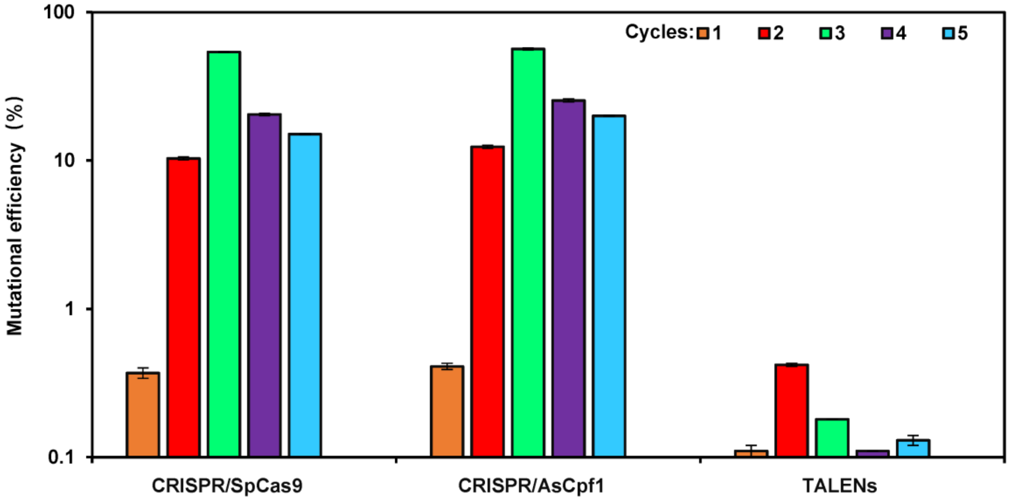

Figure S9. Improving the mutational efficiency of CRISPR/SpCas9, CRISPR/AsCpf1 and TALENs via iterative editing. *S. cerevisiae* BY4741a was transformed with p423-gRNA(URA3-1)-SpCas9, p423-crRNA(URA3)-AsCpf1 and p423-GAL-L12R12, respectively, to evaluate the mutational efficiency of CRISPR/SpCas9, CRISPR/AsCpf1 and TALENs editing tool after the different iterative cycle. The initial yeast cell population was first cultured in SD media containing 2% glucose and the auxotrophic compounds for 12h, and then was transferred into the SD media containing 2% galactose and the auxotrophic compounds for 24h to initiate the process of editing. The cell population was iteratively edited and plated onto SD+5-FOA media for evaluating the mutational efficiency by plate count method as described in Figure S2.

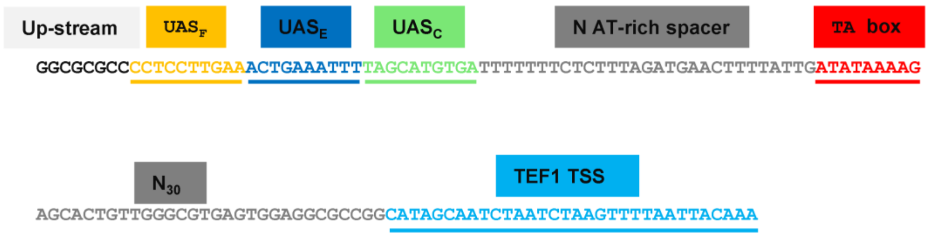

Figure S10. The DNA sequence of the synthetic minimal promoter *Pmini*. The draft of the synthetic minimal promoter used for driving *eGFP* expression has been shown in detail. Three different synthetic *UAS* elements (UAS_F, UAS_E and UAS_C) are positioned in turn at the upstream of the core TATA box and separated using a singular AT-rich neutral 30 bp spacer. Following that, oligonucleotides of 30bp (N30) was placed between TATA box and the *TEF1* transcription start site (TSS).

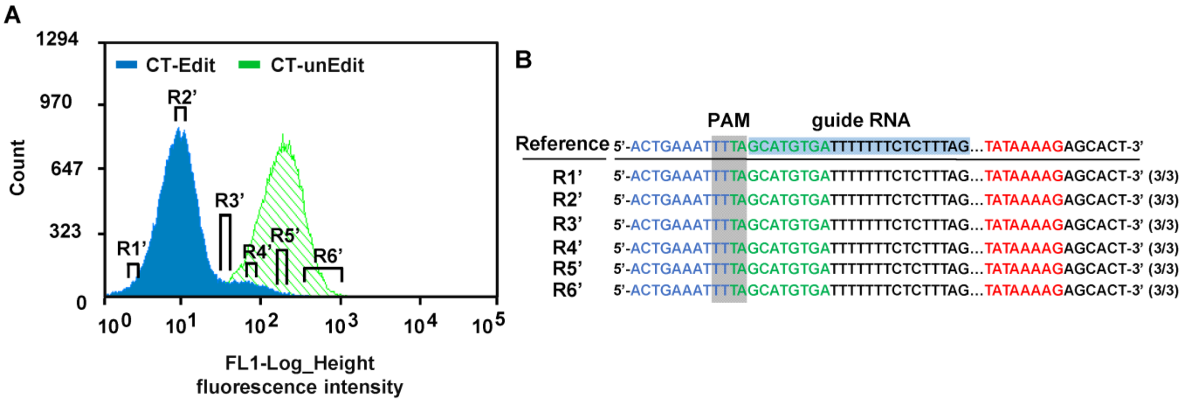

Figure S11. Mutational diversity of the fluorescence expression and genome mutagenesis resulted from the synthetic promoter editing via mGE in wild type. (A) The distribution of fluorescence of mGE-based edited control populations (CT-Edit). (B) Three distinct random clones of the six phenotypic diversity ranges were sorted out from iterative mGE-based edited populations (CT-Edit, R1'-R6') and analyzed by DNA sequencing to align their genetic variations.

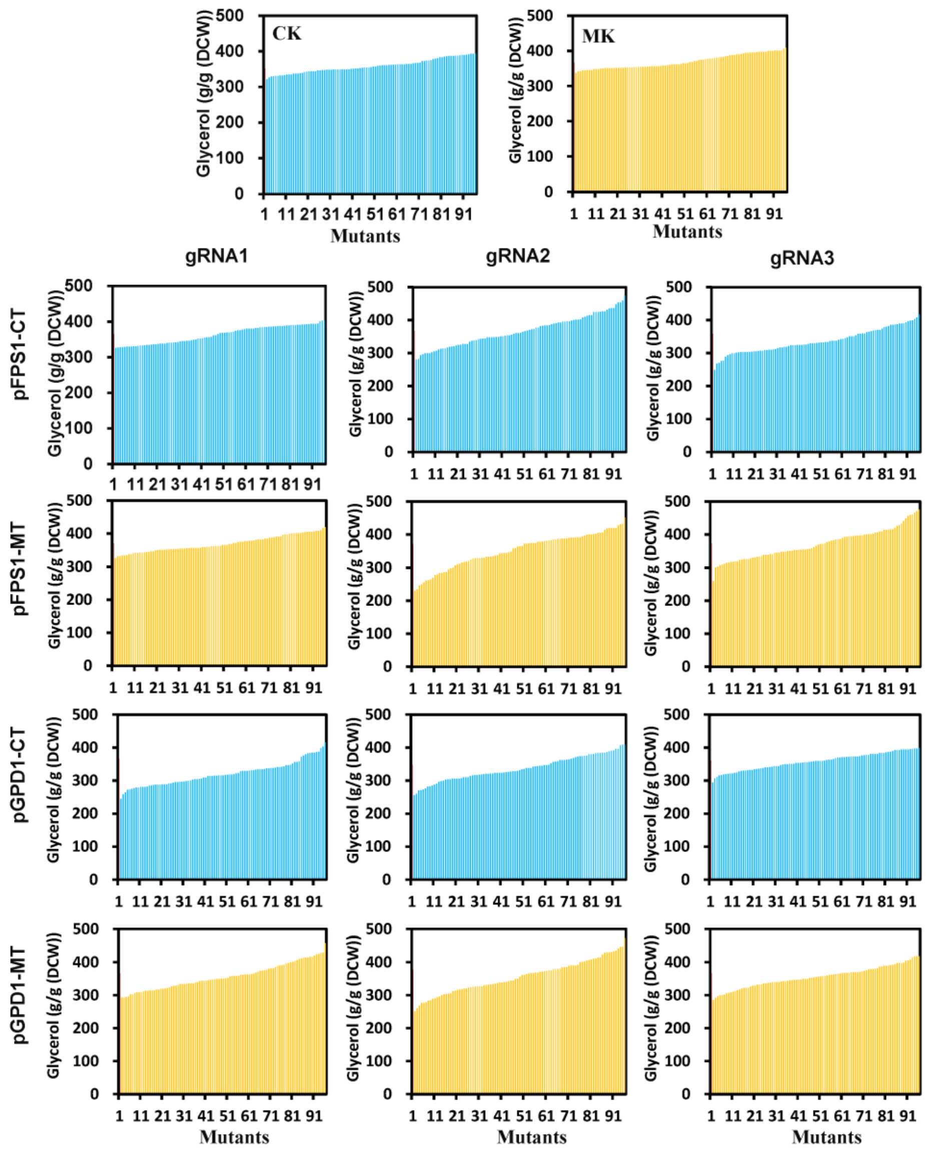

Figure S12. Characterization of the diversity of glycerol productivity resulted from pFPS1 and pGPD1 editing via mGE with three guides. Glycerol productivity of MT-Edit and CT-Edit populations were examined in ethanol fermentation. Briefly, after three cycles of iterative editing, each 100 mutants generated by CRISPR/AsCpf1-mediated mGE were randomly selected from eighteen populations of pFPS1-CT, pFPS1-MT, pGPD1-CT, pGPD1-MT (gRNA1, gRNA2 and gRNA3: different guides). Red bar: Unedited parent was used as the control. CK: BY4741 contains empty plasmids pRS423 and pRS315 without editing modules; MK: *mre11*Δ contains empty plasmids pRS423 and pmre-H125N without editing module; CK and MK were used as the control to evaluate population diversity.

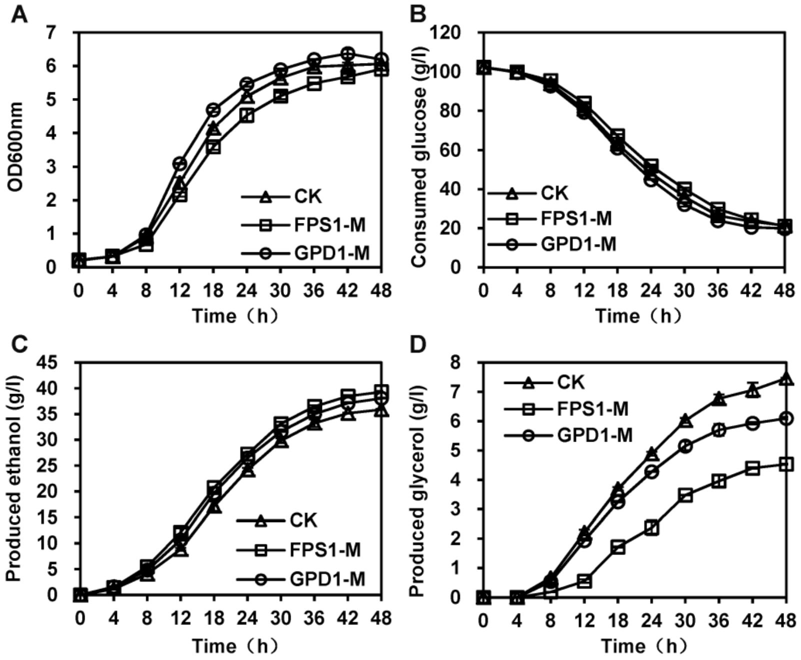

Figure S13. Evaluation of the fermentation properties of FPS1-M and GPD1-M. Characterization of glycerol and ethanol yield of three distinct mutants and the control strain. (A) Cell growth, (B) Consumed glucose, (C) Produced ethanol and (D) Produced glycerol were evaluated. Ethanol fermentation experiment was performed at 30ºC using SD media containing 100 g/l glucose and appropriate auxotrophic compounds. Initial OD_{600} of 0.2 was used for all the fermentations. The data represent the mean and standard error of the duplicate cultures under each condition.

Dataset_S1.xls

(This dataset lists mutation types at the *URA3* loci in wild type and various DSB repair mutant strains generated by four genome editing tools form 100 random sequencing events)

Mutational landscape and frequency of each edited mutant and wild type strain (Figure 3) after different genome editing were characterized. For either edited mutant or wild type strain, each 100 randomly picked out 5-FOA-resistant colonies as described in Figure 2 were selected for sequencing the target *URA3* locus, and total mutation events based on sequencing information were analyzed and grouped as >11-nt deletion, 2~11-nt deletion, 1-nt deletion, 1-nt insertion, multiple-nt insertion and multiple mismatch. The ratio of each group was also calculated.

Dataset_S2.xls

(This dataset lists mutation types at *URA3*, *ADE2*, pFPS1 and pGPD1 detected by amplicon sequencing)

Mutational landscape and frequency of 14 populations (Figure S7) and 12 populations (Figure 7) were characterized. Amplicon sequencing was employed using Illumina NovaSeq 6000 to obtain high quality reads, and then MUSCLE (Version 3.8.31) and DNA MAN (version 5.2) were performed for multiple alignment and identifying genomic variants.

Dataset_S3.xls

(This dataset lists the primers used in this study)

Dataset_S4.xls

(This dataset lists the strains used in this study)

REFERENCES

1. Zhang G, Lin Y, Qi X, Li L, Wang Q, Ma Y. TALENs-Assisted Multiplex Editing for Accelerated Genome Evolution To Improve Yeast Phenotypes. *ACS Synth Biol* **4**, 1101-1111 (2015).

2. Ryan OW, *et al.* Selection of chromosomal DNA libraries using a multiplex CRISPR system. *Elife* **3**, (2014).

3. Zetsche B, *et al.* Cpf1 is a single RNA-guided endonuclease of a class 2 CRISPR-Cas system. *Cell* **163**, 759-771 (2015).

4. Sadowski I, Lourenco P, Parent J. Dominant marker vectors for selecting yeast mating products. *Yeast* **25**, 595-599 (2008).
